# Cost-effectiveness analysis of typhoid conjugate vaccines in an outbreak setting: a modeling study

**DOI:** 10.1186/s12879-023-08105-2

**Published:** 2023-03-08

**Authors:** Maile T. Phillips, Marina Antillon, Joke Bilcke, Naor Bar-Zeev, Fumbani Limani, Frédéric Debellut, Clint Pecenka, Kathleen M. Neuzil, Melita A. Gordon, Deus Thindwa, A. David Paltiel, Reza Yaesoubi, Virginia E. Pitzer

**Affiliations:** 1 Department of Epidemiology of Microbial Diseases, Yale School of Public Health, 60 College St., P.O. Box 208034, New Haven, CT 06520-8034 USA; 2 Swiss Tropical and Public Health Institute, Basel, Switzerland; 3 Center for Health Economics Research and Modeling Infectious Diseases, University of Antwerp, Antwerp, Belgium; 4 International Vaccine Access Center, Department of International Health, Bloomberg School of Public Health, Johns Hopkins University, Baltimore, MD USA; 5 Malawi Liverpool Wellcome Programme, Blantyre, Malawi; 6 Kamuzu University of Health Sciences, Blantyre, Malawi; 7 Center for Vaccine Innovation and Access, PATH, Geneva, Switzerland; 8 Center for Vaccine Innovation and Access, PATH, Seattle, WA USA; 9 Center for Vaccine Development, University of Maryland School of Medicine, Baltimore, MD USA; 10 Institute of Infection, Veterinary and Ecological Sciences, University of Liverpool, Liverpool, UK; 11 Department of Infectious Disease Epidemiology, London School of Hygiene and Tropical Medicine, London, UK; 12 Department of Health Policy, Yale School of Public Health, New Haven, CT USA

**Keywords:** Typhoid fever, Reactive vaccination, Preventive vaccination, Typhoid conjugate vaccines, Economic evaluation

## Abstract

**Background:**

Several prolonged typhoid fever epidemics have been reported since 2010 throughout eastern and southern Africa, including Malawi, caused by multidrug-resistant *Salmonella* Typhi. The World Health Organization recommends the use of typhoid conjugate vaccines (TCVs) in outbreak settings; however, current data are limited on how and when TCVs might be introduced in response to outbreaks.

**Methodology:**

We developed a stochastic model of typhoid transmission fitted to data from Queen Elizabeth Central Hospital in Blantyre, Malawi from January 1996 to February 2015. We used the model to evaluate the cost-effectiveness of vaccination strategies over a 10-year time horizon in three scenarios: (1) when an outbreak is likely to occur; (2) when an outbreak is unlikely to occur within the next ten years; and (3) when an outbreak has already occurred and is unlikely to occur again. We considered three vaccination strategies compared to the status quo of no vaccination: (a) preventative routine vaccination at 9 months of age; (b) preventative routine vaccination plus a catch-up campaign to 15 years of age; and (c) reactive vaccination with a catch-up campaign to age 15 (for Scenario 1). We also explored variations in outbreak definitions, delays in implementation of reactive vaccination, and the timing of preventive vaccination relative to the outbreak.

**Results:**

Assuming an outbreak occurs within 10 years, we estimated that the various vaccination strategies would prevent a median of 15–60% of disability-adjusted life-years (DALYs). Reactive vaccination was the preferred strategy for WTP values of $0–300 per DALY averted. For WTP values > $300, introduction of preventative routine TCV immunization with a catch-up campaign was the preferred strategy. Routine vaccination with a catch-up campaign was cost-effective for WTP values above $890 per DALY averted if no outbreak occurs and > $140 per DALY averted if implemented after the outbreak has already occurred.

**Conclusions:**

Countries for which the spread of antimicrobial resistance is likely to lead to outbreaks of typhoid fever should consider TCV introduction. Reactive vaccination can be a cost-effective strategy, but only if delays in vaccine deployment are minimal; otherwise, introduction of preventive routine immunization with a catch-up campaign is the preferred strategy.

**Supplementary Information:**

The online version contains supplementary material available at 10.1186/s12879-023-08105-2.

## Introduction

Typhoid fever is a major source of morbidity and mortality in developing countries. Approximately 10–20 million cases and 100,000–200,000 deaths are attributed to typhoid fever each year [1, 2], accounting for 3.86–13.90 million disability-adjusted life years (DALYs) [2, 3]. Since 2010, there have been several prolonged typhoid outbreaks in eastern and southern Africa, which have imposed considerable costs to the populations impacted [4–7]. These outbreaks are associated with the emergence of antimicrobial-resistant (AMR) strains and, as a result, more outbreaks are likely as resistance spreads [4, 7, 8].

Typhoid conjugate vaccines (TCVs) are an effective means of typhoid prevention and control with the goal of reducing the burden and spread of typhoid fever [9–11]. They have been approved and recommended by the World Health Organization (WHO) and Gavi (the Vaccine Alliance) has pledged support for introduction of TCVs in typhoid-endemic countries. At its April 2022 meeting, the WHO Strategic Advisory Group of Experts on Immunization (SAGE), acknowledged new data demonstrating high efficacy and effectiveness of a single dose of TCV across diverse settings and reaffirmed the current recommendations for TCV use. Likewise, limited data from Harare, Zimbabwe support declines in typhoid cases following TCV use for outbreak control [12]. The vaccine’s short-term efficacy against infection and disease has been shown [9–11], but research regarding its longer-term efficacy and role in reducing transmission is still underway [9, 11]. Current data are limited on how and when TCVs should be introduced, and TCV stockpiles do not yet exist [13, 14].

Reactive vaccination has become an important prevention measure for outbreaks of diseases such as cholera, influenza, and Ebola [15–22]. While reactive vaccination can be effective, the impact will be small if implemented late or focused inappropriately on less vulnerable populations [15, 19, 20]. Although TCVs are recommended by the WHO for use in outbreak settings, there is no specific guidance on how to define the start of an outbreak of typhoid fever in order to trigger a reactive vaccination response. Furthermore, policymakers are faced with inevitable delays in securing TCVs for outbreak response and applying for Gavi support to implement routine vaccination. While preventative introduction of TCVs in low-incidence settings is unlikely to be cost-effective in the absence of outbreaks [23, 24], it may help to prevent future outbreaks of typhoid fever associated with introduction of AMR strains. Hence, it is necessary to evaluate the cost-effectiveness of preventative versus reactive vaccination strategies in the presence and absence of outbreaks of typhoid fever.

Here, we use a dynamic transmission model fitted to data from a typhoid fever outbreak in Blantyre, Malawi to investigate the health impact, costs, and cost-effectiveness of alternative vaccine delivery strategies and to inform the use of TCVs in an outbreak setting. We explored a range of preventative and reactive vaccination scenarios to examine the robustness of our findings in the face of uncertainty in outbreak timing, outbreak identification, and delays in vaccine introduction.

## Methods

### Transmission model and outbreak threshold

We developed an age-specific stochastic model to simulate typhoid fever transmission dynamics in Blantyre, Malawi from January 1995 to December 2031. The time-horizon includes a long pre-outbreak period, the outbreak itself (which peaked in 2013), and a sufficiently long post-outbreak period to account for relevant population-level impacts of vaccination. Details of the model are provided in the Additional file 1. The model was parameterized based on the equivalent deterministic model fitted to routine blood-culture surveillance data from Queen Elizabeth Central Hospital (QECH) in Blantyre from January 1996 to February 2015 [8], which captures the multi-year outbreak of typhoid fever that occurred in Blantyre between 2011 and 2015. We assumed the outbreak was caused by an increase in the duration of infectiousness of *Salmonella* Typhi associated with the emergence of the multidrug-resistant H58 haplotype [8, 25]. We estimated the pre-outbreak basic reproductive number (*R*_0_), timing and magnitude of increase in the duration of infectiousness, amplitude of seasonality in transmission, and relative infectiousness of chronic carriers by fitting to age-stratified data on the number of weekly culture-confirmed typhoid fever cases at QECH; the remaining model parameters were fixed based on data from the literature (see Table 1). We validated the model by comparing it to blood-culture surveillance data from QECH for March 2015 to December 2016. To scale the number of blood-culture-confirmed cases at QECH to the population-based incidence of typhoid fever in Blantyre, we used data from the recently completed Strategic Typhoid Alliance across Africa and Asia (STRATAA) cohort study (Additional file 1: S1.1.4 Text) [26].

**Table 1:** Dynamic model input parameters

Characteristic	Value	Source
*Demographic parameters*		
Crude birth rate (*B*)	31.3–55.0 live births per 1000 per year, based on estimates for 1950–2035	[27]
Crude death rate ($\mu$)	7.7–27.8 deaths per 1000 per year, based on estimates for 1950–2035^a^	[27]
*Disease parameters*		
Duration of infectiousness ($1/\delta$) (pre-outbreak)	3 weeks	[8, 28] Assumes infections can be effectively treated with antibiotics
Seasonal offset parameter (timing of seasonal peak) ($\phi$)	4.9 weeks (early February)	[8] Based on timing of peak rainfall in Blantyre
Fraction infected who become chronic carriers ($\theta$)	0.003–0.101 depending on age	[29] We assume only first infections lead to chronic carriage
Disease-induced mortality ($\alpha$)	0.001	[8, 30]
Duration of temporary full immunity to infection ($1/\omega$)	104 weeks	[8, 28]
Pre-outbreak basic reproductive number (*R*_*0*_)	3.29	Refit parameters from modified Pitzer et al. model [8]
Amplitude of seasonal forcing (*q*)	0.35	Refit parameters from modified Pitzer et al. model [8]
Relative infectiousness of chronic carriers (*r*)	0.09	Refit parameters from modified Pitzer et al. model [8]
*Outbreak parameters*^*b*^		
Beginning week of increase in duration of infectiousness (*t*_*1*_)	April 10, 2011	Refit parameters from modified Pitzer et al. model [8]
End week of increase in duration of infectiousness (*t*_*2*_)	November 23, 2014	Refit parameters from modified Pitzer et al. model [8]
Magnitude of increase in duration of infectiousness (*m*)	3.1954	Refit parameters from modified Pitzer et al. model [8]
*Reporting process*		
Underreporting adjustment factor (*a*)	7.7 (95% CrI: 6.0–12.4)	[26]
*Vaccine-related parameters*		
Age groups vaccinated		Based on WHO recommendation
Routine	9 months	
Catch-up campaign	9 months to < 15 years	
Initial efficacy of TCV against infection (${\nu }_{0}$)	0.89 (95% CrI: 0.78–0.98)	Re-analysis based on Malawi TCV efficacy trial data and a previous estimate from [9, 23] (Additional file 1: S1.1.2.2. Text, Fig. S2)
Average duration of vaccine-induced immunity ($1/{\omega }_{v})$	Vaccine efficacy decreases exponentially with an average duration of 18.9 (95% CrI: 8.4–83.3) years	Re-analysis based on Malawi TCV efficacy trial data and a previous estimate from [9, 23] (Additional file 1: S1.1.2.2. Text, Fig. S2)
Vaccine coverage		Gavi demand forecasts under assumption of unconstrained supply, and commonly assumed coverage during a catch-up campaign during an outbreak
Routine (${\kappa }_{R}$)	Increases from 0.85 to 0.95 over ten years	
Catch-up campaign (${\kappa }_{C}$)	Uniform (0.6,0.9)	

Demographic, disease, outbreak, reporting, and vaccine parameters used in the dynamic transmission model are shown. Demographic, disease, and outbreak parameters used a combination of pre-defined values from a previous model and numbers estimated through the re-calibrated deterministic version of the model, noted in the “Source” column. *CrI*  credible interval

^a^Further adjusted to account for migration and reproduce population size and age distribution between 1999 and 2011

^b^Assumed a linear increase in the duration of infectiousness between t_1_ and t_2_ from the pre-outbreak value of 3 weeks to a final value of 3*m weeks, due to the emergence of multidrug resistance

Stochastic model projections of the number of typhoid fever cases through time incorporated uncertainty in the transmission dynamics using a Poisson process for each transition between states and a binomial observation process for the (under)reporting of cases over the duration of an individual’s infection. To simulate the impact of vaccination, we incorporated further uncertainty in vaccine efficacy at time 0, the waning of vaccine-induced immunity, and vaccine coverage during the catch-up campaign by sampling from the associated uncertainty distribution for each stochastic iteration (Additional file 1: S1 Text). We assumed vaccination protects against both infection and disease. The dynamic model parameters, their estimates and uncertainty distributions, and sources are listed in Table 1. When possible, we used parameter values estimated in previous studies, noted in Table 1. For other parameters, the estimation methods are unique to each parameter and are described in more detail in the supplement. For each intervention strategy, we simulated the outbreak 1000 times.

Since there is no globally-defined threshold for a typhoid fever outbreak, we explored different definitions of the epidemic threshold. For the purposes of our analysis and to facilitate outbreak identification from passive hospital-based surveillance data across different populations and contexts, we specified the epidemic threshold in terms of the number of standard deviations (SD) above the mean monthly reported typhoid fever cases for the baseline period of 2000–2010. We examined thresholds ranging from 6 to 16 SD above the mean, and defined the “true” start of the outbreak as April 10, 2011, identified during model-fitting. For reference, this corresponded to a range of approximately 10–25 typhoid fever cases per month above the monthly mean of approximately 1.4 cases per month in this setting. Setting a threshold too low would trigger too many false positive identifications of the outbreak, while setting the threshold too high could fail to identify a true outbreak in a timely manner. To address this issue, we compared the sensitivity and specificity of each outbreak definition. We defined the sensitivity of each threshold as the percentage of simulations in which the outbreak was identified within 18 months of April 2011, while the specificity was defined as the percentage of simulations in which the outbreak threshold was not exceeded prior to April 2011. Once the outbreak threshold was crossed, all subsequent months were considered to be part of the outbreak. For our primary analysis, we used the outbreak identification threshold that yielded the highest sum of sensitivity and specificity.

### Vaccination scenarios

We considered three scenarios under the assumption that a country has not yet implemented routine vaccination: (1) an outbreak is likely to occur over the next 10 years; (2) an outbreak is unlikely to occur (and hence, the country maintains the same pre-outbreak seasonal incidence); and (3) an outbreak has already occurred and is unlikely to happen again (and hence, the country has a higher incidence post-outbreak compared to pre-outbreak). The most likely scenario depends on the recent history of typhoid incidence and that of nearby regions. If surrounding regions have been experiencing outbreaks but the country has not yet had one, we assume that it is likely that an outbreak will occur at some point within the next 10 years (Scenario 1). For this scenario, we randomized the timing of the start of the outbreak to follow a uniform distribution over Years 0–10; we assumed only a single outbreak occurs. However, if surrounding regions are not experiencing outbreaks, it may be unlikely that an outbreak would occur (Scenario 2). If an outbreak has already occurred, as it did in Malawi, we assume another outbreak is unlikely within the next 10 years (Scenario 3).

Because the outbreak in Malawi was driven by the emergence of multi-drug resistance, typhoid incidence under the Scenario 3 (post-outbreak) is higher than the incidence under Scenario 2 (pre-outbreak). For Scenario 2, we assume typhoid fever incidence is comparable to that estimated for Blantyre for 1995–2005, whereas for Scenario 3, we assume it is comparable to that predicted for Blantyre for 2021–2031. These scenarios are comparable to previous cost-effectiveness analyses and allow us to examine whether it would be beneficial to introduce TCV in an endemic setting when typhoid fever incidence is lower (Scenario 2: pre-outbreak) or higher (Scenario 3: post-outbreak).

We simulated four alternative vaccination strategies, following previous cost-effectiveness analyses of TCV strategies and the current WHO recommendation in endemic settings [14, 23, 24, 31]: no vaccination (base case), preventive routine TCV introduction at 9 months of age (in Year 0), preventive routine vaccination plus a one-time catch-up to age 15 (also in Year 0), and (for Scenario 1 only) reactive routine vaccination plus a catch-up campaign to age 15 once the outbreak was identified (Table 2). Vaccine efficacy parameters (including the initial vaccine efficacy and exponential rate of waning immunity) were estimated by fitting to data from a phase 3, double-blind, randomized active-controlled clinical trial of single-dose Typbar TCV in Blantyre, Malawi [9, 23] (Table 1, Additional file 1: S1.1.2.2 Text, Fig. S2). Routine vaccination coverage was assumed to increase from 85 to 95% over the first ten years of vaccination and then remain at 95% [23]. For catch-up campaign coverage, we varied the proportion vaccinated uniformly from 60 to 90%.

**Table 2:** Strategy comparisons for deploying typhoid conjugate vaccines to prevent or respond to an outbreak

Strategy type	Vaccination strategies
Base	No vaccination
Preventive	Routine at 9 months
Preventive	Routine + catch-up to age 15
Reactive	Routine + catch-up to age 15

Each of the scenarios examined compares four strategies: a base case (no vaccination), a preventive strategy with routine vaccination at 9 months of age (“routine”), a preventive strategy with routine vaccination and a catch-up campaign up to 15 years of age (“routine + catch-up”), and a reactive vaccination strategy with routine vaccination and a catch-up campaign

### Economic evaluation

To measure disease burden and hence identify vaccination strategies that most effectively reduce the burden of typhoid fever in this setting, we calculated disability-adjusted life-years (DALYs) due to typhoid fever. The stochastic model output for the number of typhoid fever cases and vaccine doses administered per 100,000 people under each strategy was used to calculate the DALYs due to typhoid, costs of treatment, and costs of vaccine delivery. Costs of vaccination programs are generally incurred by the government and donors in Malawi; hence, we considered the healthcare system perspective and only accounted for direct treatment and vaccination costs accrued by the healthcare system. Costs were converted to 2020 USD. We conducted the analysis in accordance with WHO guidelines and recommendations of the Bill and Melinda Gates Foundation’s reference case [32–35]. All costs and effects were discounted at a rate of 3% per year. The analysis adheres to the Consolidated Health Economic Evaluation Reporting Standards (CHEERS), where applicable [32] (Additional file 1: S3 Text).

Consistent with WHO guidelines and recommendations of the Bill and Melinda Gates Foundation’s reference case [32–35], we evaluated the effectiveness of each strategy in terms of DALYs. DALYs represent the total years of life lost due to death (YLL) and lived with disability (YLD) due to the disease: $DALY=YLL+YLD$ [36]. To estimate the YLLs due to typhoid fever, we multiplied the number of cases of typhoid fever by the probability of hospitalization and the probability of death for inpatients (Additional file 1: S1.2.6 Text) [37, 38]. We then divided by the proportion of deaths occurring in hospital, which we assumed was uniformly distributed between 0.25–1 [23], to obtain an estimate of the total number of deaths, and subtracted the average age of death from typhoid fever from Malawi’s life expectancy [39]. The YLDs were calculated based on the number of cases, duration of illness, and disability weights (Additional file 1: S1.2.3 Text).

To estimate the treatment costs for typhoid fever, we assumed 71% (95% CrI: 64–77%) of typhoid fever cases would seek medical care and 4% (95% CrI: 1–11%) would be hospitalized; we updated prior distributions for these parameters based on data from the STRATAA cohort study [26] (Table 3; Additional file 1: S1.2.4–1.2.5 Texts). The number of outpatient cases was calculated by subtracting the number of hospitalized cases from the number of individuals seeking care. We assumed cases not seeking medical care would not incur treatment costs. We estimated treatment costs for typhoid fever using a recent cohort study on treatment costs for typhoid fever in Blantyre, Malawi [40].

**Table 3:** Input parameters for cost-effectiveness analysis

Characteristic	Median value (95% CrI)	Source
*Typhoid incidence and age distribution*		
Annual number of symptomatic typhoid fever cases per 100,000 people (without vaccination)	26.1 (14.0–45.7) before outbreak; Up to 916 (823–1543) during outbreak; 224 (169–368) after outbreak	Based on output from transmission dynamic model fitted to culture-confirmed cases and population-based adjusted incidence
Average age of patients with typhoid infection (without vaccination) (years)	15.9 (13.8–19.3)	Based on output from transmission dynamic model fitted to culture-confirmed cases
*Typhoid mortality*		
Probability of death if patients are admitted to hospital for typhoid infection	0.09 (0.02–0.28)	[38, 41] [42]
Proportion of deaths from typhoid infection occurring in patients not hospitalized	0.38 (0.02–0.73)	Assuming that on average about one of three deaths occur outside the hospital setting, from [23]
Average age at death from typhoid infection (years)	15.9 (13.8–19.3)	Assuming age distribution of deaths is the same as the age distribution of patients with typhoid
*Antimicrobial resistance*		
Proportion of patients with typhoid infection with an AMR strain	0.001 (0.00–0.63) before outbreak; Up to 0.96 (0.86–1.00) during outbreak; 0.65 (0.31–0.98) after outbreak	[25, 42]
Burden of AMR cases relative to antimicrobial-sensitive cases	2 (1–3)	[23]
*Healthcare use*		
Probability of infected patients seeking healthcare	0.71 (0.64–0.77)	[26]
Probability that infected patients are admitted to hospital	0.04 (0.01–0.11)	[43] [42]
Length of stay in hospital (days)	6 (3–9)	[23]
*Treatment costs*		
Cost of inpatient treatment	$214.38 ($164.41–$264.34)	[40]
Cost of outpatient treatment	$39.67 ($33.96–$45.39)	[40]
Cost of treatment for a patient not seeking professional medical care	$1.59 ($0.31–$2.86)	[40]
*Vaccine-related costs*		
Vaccine procurement cost per dose (accounting for Gavi support)	Routine: $0.20 Campaign: $0.00	Assuming Gavi support and that Malawi remains in the initial self-financing phase
Injection and safety equipment	$0.23 ($0.21–$0.24)	[23]
Vaccine delivery cost per dose (accounting for Gavi support)	Routine^a^: $1.61 ($0.36–$4.23) Campaign: $0.40 ($0.23–$0.62)	Based on a meta-analysis of delivery costs for new vaccine introductions; see Bilcke et al. for details [23]
Number of years during which start-up costs of vaccine delivery program are incurred	2 (1–3)	[23]
Percent of routine vaccine delivery costs that are ongoing	64% (48–78%)	[23]
*Disability-adjusted life-years*		
Disability-weights (from 0 = perfect health to 1 = death)	Severe illness, 0.21 (0.14–0.29); moderate illness, 0.052 (0.031–0.079); mild illness, 0.005 (0.002–0.011)	[44]; see Additional file 1: S1.2.3 for explanation of how disability weights were assigned to different healthcare use groups
Duration of illness in inpatients and outpatients (days)	16 (12–20)	[23]
Relative duration of illness for patients not seeking medical care (vs inpatients and outpatients)	0.5 (0.02–0.98)	[23]
Life expectancy (years)	62.7	[39]

^a^Does not account for Gavi Vaccine Introduction Grant support of up to $0.80 per dose in the first year

Antimicrobial resistance is likely to affect the burden and costs associated with typhoid fever. In the absence of sufficient data to parameterize the relative burden and costs of AMR typhoid fever, we assumed the case fatality risk, years of life lived with disability, and treatment costs were twice as high on average (uniformly 1–3 times higher) for AMR cases. Since recent outbreaks are thought to be caused by new AMR strains of typhoid, we allowed the proportion of AMR typhoid cases to vary with time based on data from a longitudinal study in Blantyre (Additional file 1: S1.2.7 Text) [25].

In our analysis, we included only the costs to be paid by the government of Malawi (i.e. accounting for Gavi support), assuming Malawi remains in the initial self-financing phase of Gavi support. Thus, we assumed a vaccine procurement cost of $0.20 per routine dose and $0 for campaign doses ($1.50/dose prior to Gavi support) (Table 3; Additional file 1: S1.2.2 Text). We also accounted for Gavi support for the delivery of routine doses in the first year of vaccination ($0.80/dose). All costs were adjusted for inflation to 2020 USD.

### Sensitivity analyses

To assess the robustness of our economic evaluation to the underlying parameter uncertainty, we accounted for uncertainty with probabilistic sensitivity analysis and explored its impact in two ways: (1) we examined cost-effectiveness acceptability frontiers to assess how parameter uncertainty contributes to uncertainty in the optimal strategy; (2) value of information analysis to identify the most influential parameters and to inform if it is worth delaying the decision in anticipation of better information at a later date, by estimating the expected value of partially perfect information (EVPPI) for each parameter with the one-level method devised by Strong and Oakley [45].

We randomly drew 5000 independent samples from the uncertainty distributions of each input parameter in the economic evaluation (Additional file 1: Table S2). Each sample was combined with one of the samples from the stochastic transmission model (1000 simulations repeated five times to achieve a manageable computational burden) to estimate 5000 net monetary benefit (NMB) values for each strategy and for a range of willingness-to-pay (WTP) values from $0–$1000 in increments of $10. The NMB is calculated as $$NMB=\Delta E*WTP-\Delta C$$, where $$\Delta E$$ is the DALYs averted by each vaccination strategy compared to the base case of no vaccination, $$\Delta C$$ is the incremental cost of the vaccination strategy compared to the base case, and *WTP* is the willingness-to-pay threshold. We quantified the uncertainty surrounding the optimal strategy by calculating the proportion of samples for which a strategy yielded the highest NMB for each WTP. We also measured the contribution of each input parameter to the uncertainty around the optimal vaccination strategy by calculating the EVPPI.

### Scenario analyses

While countries have the option of introducing TCVs into the routine immunization program, to date no vaccine stockpile exists for TCV introduction in the event of an outbreak.

To address this uncertainty, we account for varying delays in reactive vaccine deployment. For our primary analysis, we assumed an “idealized” scenario in which vaccination is introduced within 1 month of identifying the outbreak. In scenario analyses, we explored deployment delays of 6, 12, and 24 months after the epidemic threshold was exceeded.

The optimal strategy may also depend on how long until the outbreak occurs. We examined scenarios in which TCV introduction occurs exactly 10 years or 1 year before the epidemic threshold is crossed. For this comparison, we assessed the burden of typhoid fever and costs of treatment and vaccination for the preventative and reactive vaccination scenarios over a 20-year time horizon spanning from 2000 to 2020. For all other analyses, we used the same 10-year time horizon to match previous cost-effective analyses.

A previous cost-effectiveness analysis of TCVs used WHO-CHOICE data for cost-of-illness estimates [23, 46]. Since the Malawi-specific cost-of-illness estimates used in this analysis were higher, we additionally evaluated the cost-effectiveness of the idealized scenario using the previous WHO-CHOICE cost-of-illness estimates.

The stochastic transmission model and economic model were implemented in R version 3.4.0 [47]. The transmission model code is available on GitHub at https://github.com/mailephillips/typhoid_outbreak_Malawi.

## Results

The model accurately reproduced the number and age distribution of observed blood-culture confirmed typhoid fever cases at QECH during both the fitting period (January 1996–February 2015) and the validation period (March 2015–December 2016) (Additional file 1: Figs. S5, S6). Over the 10-year simulation period with randomized outbreak timing (Scenario 1), we estimated a median of 1989 (95% CrI: 210–3508) cases and 18 (95% CrI: 1–145) deaths per 100,000 people for a total of 398 (95% CrI: 21–3109) DALYs and $126,754 (95% CrI: 9267–290,227) in treatment costs per 100,000 people for typhoid fever under the no vaccination strategy (Additional file 1: Table S3).

All vaccination strategies substantially reduced the expected number of typhoid fever cases, but did not completely prevent an outbreak from occurring. Preventive routine vaccination with a catch-up campaign delayed the start of the outbreak and reduced typhoid fever incidence substantially more than routine vaccination alone. When reactive vaccination was deployed within 1 to 6 months of the outbreak threshold being crossed, the epidemic was substantially smaller and delayed by 1–2 years (Additional file 1: Fig S8). However, when reactive vaccination occurred 12 to 24 months after the outbreak was identified, it failed to prevent the peak in typhoid fever cases, although incidence was substantially reduced after vaccine deployment. The number of typhoid fever cases, deaths, and DALYs averted by each vaccination strategy compared to no vaccination, as well as the associated costs, are detailed in Additional file 1: Table S4.

**Fig. 1:**
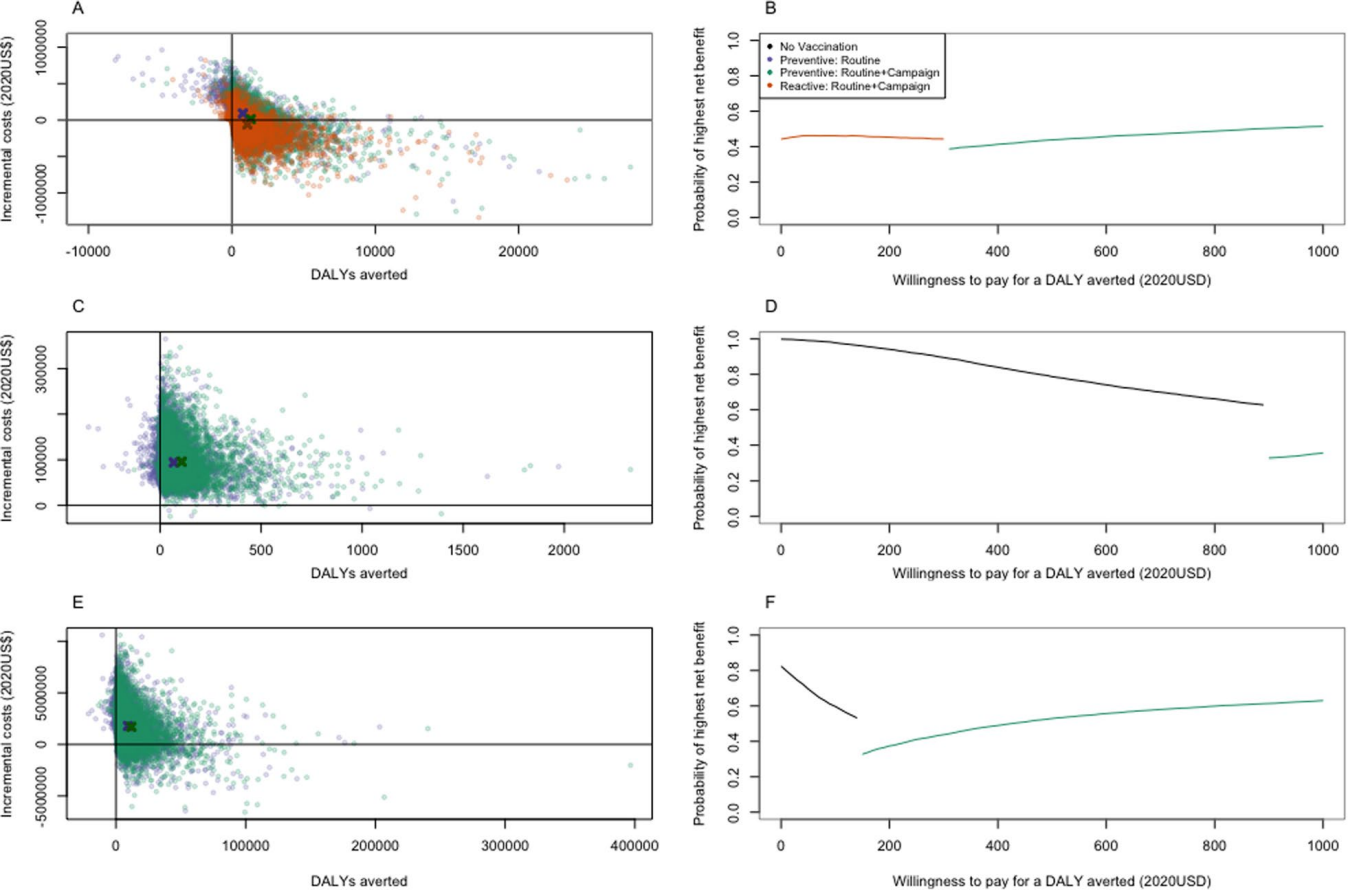
Cost-effectiveness planes and acceptability frontiers. The cost-effectiveness planes (left) and cost-effectiveness acceptability frontiers (CEAFs; right) are plotted for **A**, **B** Scenario 1 (randomized outbreak timing), **C**, **D** Scenario 2 (no outbreak), and **E**, **F** Scenario 3 (outbreak has already occurred). In the cost-effectiveness planes, each dot represents the incremental costs (in 2020 USD) and DALYs averted for one simulation when compared with the base case of no vaccination. The bold Xs denote the expected additional cost and DALYs averted for each vaccination strategy with respect to no vaccination. Strategies are indicated by the color of the dot or X (purple: preventive routine vaccination; green: preventive routine vaccination plus a catch-up campaign up to age 15; or orange: reactive routine vaccination plus a catch-up campaign to age 15—for Scenario 1 only). In the CEAFs, the preferred strategy (i.e. the strategy that yielded the highest average net benefit) for each willingness-to-pay threshold ($0–1000 per DALY averted; x-axis, 2020 USD) is indicated by the color of the line (black: no vaccination; and same strategy colors as other panels), while the proportion of samples in which that strategy yielded the highest net benefit is indicated by the value on the y-axis; this can be interpreted as our certainty in the optimal strategy

Under Scenario 1, our findings suggest that TCV introduction was cost-effective compared to no vaccination for all WTP values above $0 (Table 4 and Fig. 1B). Reactive vaccination (with a 1-month delay in implementation) was cost-saving, with estimated total costs averted of $17,147 (95% CrI: − $3500–$65,080) per 100,000 people. Reactive vaccination was preferred for a WTP range of $0–$307 (dominating the base case of no vaccination), whereas preventive routine vaccination with a catchup campaign was optimal for WTP values above $307. Routine vaccination including a catch-up campaign to 15 years of age was always preferred over routine vaccination alone.

**Table 4:** Expected cost-effectiveness of vaccination strategies

Strategy	Expected net costs per 100,000 people in 2020 USD	Expected total DALYs per 100,000 people	Expected incremental costs per 100,000 people versus next best non-dominated alternative	Expected DALYs averted per 100,000 people versus next best non-dominated alternative	ICER versus next best non-dominated alternative ($ per DALY averted)
*Scenario 1: When an outbreak occurs over the 10-year time horizon*					
Reactive vaccination (routine + campaign)	$111,213	383	–	–	–
No vaccination (base case)	$128,360	689	–	–	Dominated
Preventative vaccination (routine + campaign)	$131,749	316	$20,536	67	307
Preventative vaccination (routine only)	$154,148	472	–	–	Dominated
*Scenario 2: When no outbreak occurs (pre-outbreak incidence)*					
No vaccination (base case)	$5893	35	–	–	–
Preventive vaccination (routine only)	$26,704	22	–	–	Dominated
Preventative vaccination (routine + campaign)	$27,109	11	$21,216	24	902
*Scenario 3: When an outbreak has already occurred (post-outbreak incidence)*					
No vaccination (base case)	$7242	577	–	–	–
Preventive vaccination (routine + campaign)	$49,406	295	$42,164	282	150
Preventative vaccination (routine only)	$51,717	372	–	–	Dominated

Expected total net costs, total disability-adjusted life-years (DALYs), incremental costs, DALYs averted, and incremental cost-effectiveness ratios (ICERs) per 100,000 people are shown for each strategy over the 10-year time horizon when (1) an outbreak occurs over the 10-year time horizon (randomized timing; Scenario 1), (2) an outbreak does not occur (i.e. assuming the pre-outbreak incidence; Scenario 2), and (3) an outbreak has already occurred and another one is unlikely (i.e. assuming the post-outbreak incidence; Scenario 3). Strategies are sorted from lowest to highest expected total costs per 100,000 individuals. All costs and DALYs are discounted at a rate of 3% per year. Dominated strategies do not form part of the cost-effectiveness frontier, i.e. no WTP value exists for which a dominated strategy is preferred in terms of cost-effectiveness

If no outbreak occurs (Scenario 2, assuming the lower pre-outbreak incidence), no vaccination is the preferred strategy for all WTP thresholds < $902 (Fig. 1D). If the outbreak has already occurred (Scenario 3, assuming the higher post-outbreak incidence), routine vaccination with a catch-up campaign is preferred for WTP values of $150 or higher (Fig. 1F). Again, routine vaccination alone is never the preferred strategy. The number of typhoid fever cases, deaths, and DALYs averted, as well as the costs for each scenario, are presented in Additional file 1: Table S4.

**Fig. 2:**
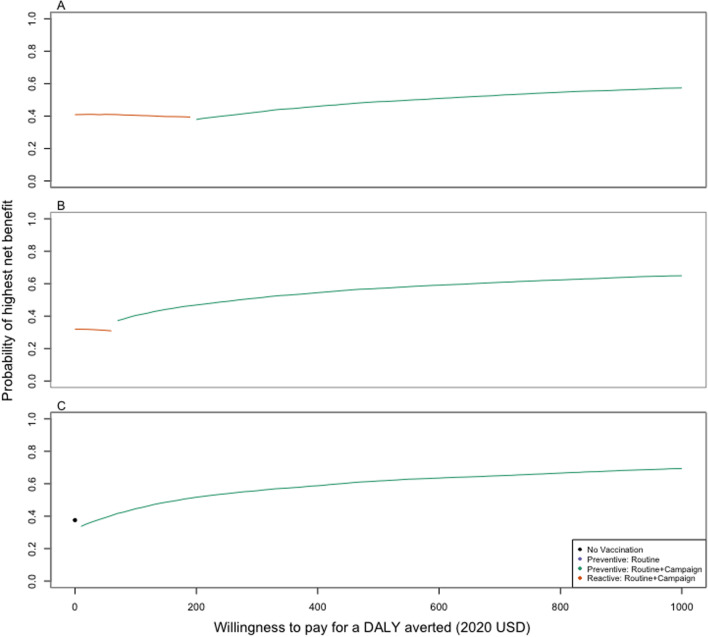
Cost-effectiveness acceptability frontiers for Scenario 1 with varying delays in reactive vaccination. The cost-effectiveness acceptability frontiers for Scenario 1 (randomized outbreak timing) are shown for a range of willingness-to-pay thresholds ($0–1000; x-axis, 2020 USD for **A** a 6-month delay in reactive vaccination after the outbreak threshold is exceeded, **B** a 12-month delay in reactive vaccination after the outbreak threshold is exceeded, and **C** a 24-month delay in reactive vaccination after the outbreak threshold is exceeded. The preferred strategy (i.e. the strategy that yielded the highest average net benefit) is indicated by the color of the line (black: no vaccination; purple: preventive routine vaccination; green: preventive routine vaccination plus a catchup campaign up to 15 years; or orange: reactive routine vaccination plus a catchup campaign), while the proportion of samples in which that strategy yielded the highest net benefit is indicated by the value on the y-axis (which can be interpreted as our certainty in the optimal strategy)

The WTP threshold at which the preferred strategy switches from reactive vaccination to preventative vaccination decreased as the delay in TCV deployment for the reactive vaccination strategy increased (Fig. 2). For a 6-month delay, reactive vaccination was the preferred strategy for WTP thresholds between $0–190 per DALY averted (Fig. 2A), while for a 12-month delay, reactive vaccination was preferred at $0–60 per DALY averted (Fig. 2B). If the delay extended up to 24 months, reactive vaccination was never preferred; preventative vaccination with a catch-up campaign was the optimal strategy when the WTP threshold was at least $10 per DALY averted; this was the only scenario in which no vaccination was ever preferred (for a WTP of $0 per DALY averted) (Fig. 2C).

The optimal vaccination strategy did not vary substantially depending on how long before the outbreak preventive vaccination was implemented. Whether the outbreak occurred within 10 years or 1 year of vaccine introduction for the preventive strategies, the preferred strategy remained essentially the same (Additional file 1: Fig S10).

When using WHO-CHOICE instead of Blantyre-specific cost of illness data, the probability that a particular vaccination strategy was preferred followed a similar pattern. However, since the WHO-CHOICE costs were lower compared to the Blantyre-specific costs, the willingness-to-pay thresholds at which different vaccination strategies were preferred was shifted approximately $100 higher (Additional file 1: Fig S11). For example, under Scenario 1 using the WHO-CHOICE cost of illness data, TCV introduction was optimal compared to no vaccination for WTP values greater than $100 (Additional file 1: Table S5 and Fig. S11B). Reactive vaccination (with a 1-month delay in implementation) was preferred for a WTP range of $110–$430, whereas preventive routine vaccination with a catch-up campaign was optimal for WTP values above $430. Routine vaccination alone was never the preferred strategy.

For all of the economic evaluations, uncertainty around the probability of death among inpatients contributed most to uncertainty in the preferred strategy, followed by the probability of hospitalization, percentage of deaths occurring among hospitalized patients, and routine vaccine delivery costs (Additional file 1: Figs. S12–S14).

## Discussion

Multi-year outbreaks of typhoid fever have occurred in numerous settings following the introduction of AMR strains. In countries where typhoid incidence is low and antimicrobial resistance is rare, but where surrounding countries in the region are experiencing outbreaks of drug-resistant typhoid fever, it is likely that AMR strains will spread, triggering further outbreaks. Our findings indicate that if an outbreak of typhoid fever is likely to occur within the next 10 years, introduction of TCVs with a catch-up campaign is likely to be cost-effective compared to no vaccination, and reactive vaccination may be cost saving if deployed within 12 months. However, if the WTP threshold is greater than $300 per DALY averted, it is generally better (in terms of cost-effectiveness) to preventively introduce routine vaccination with a catch-up campaign rather than waiting for the outbreak to occur. These findings hold true regardless of when the outbreak occurs, provided it occurs within 10 years.

As TCV stockpiles do not yet exist, there is uncertainty in how long it will take to mobilize vaccine introduction once an outbreak is identified. The cost-effectiveness of reactive vaccination largely depends on the length of delay in vaccine deployment. Decision-makers should try to realistically determine how quickly they would be able to identify a typhoid fever outbreak and mobilize resources to implement both routine TCV introduction and a catch-up campaign, and seek to minimize delays in vaccine deployment when considering reactive vaccination strategies. In settings that lack established platforms for blood culture surveillance and the resources to implement TCV campaigns within a year, preventative introduction of TCVs should be favored. Nevertheless, improved surveillance of typhoid fever is needed.

There is currently no threshold for defining and identifying outbreaks of typhoid fever across different settings. In a recent review of typhoid fever outbreaks occurring between 1989 and 2018, the number of reported cases ranged from 3 to 10,677 [48]. In our analysis, we found that an increase in the monthly number of blood-culture-confirmed typhoid fever cases of more than 15 standard deviations above the mean accurately identified the start of the outbreak in Blantyre, Malawi while avoiding false alarms due to normal seasonal variations. It is not yet clear whether this threshold may be applicable to other settings. However, the results of our analysis are unlikely to depend on the outbreak identification threshold used. While lower thresholds may falsely identify an outbreak before it occurs, a false alarm may not be problematic provided the outbreak still occurs. If the outbreak is falsely identified too early, “reactively” vaccinating in response to the false outbreak is comparable to a preventative vaccination strategy, which in our analysis was still cost-effective for WTP thresholds above $300, provided an outbreak occurs sometime within the next 10 years. Given the consequences of delaying outbreak response, it is preferable to err on the side of early (false) outbreak alarms. These findings are encouraging as typhoid resources and surveillance systems are often sub-optimal in areas where typhoid is endemic.

For scenarios in which an outbreak does not occur, our results are consistent with previous cost-effectiveness analyses, though with lower WTP thresholds where vaccination is preferred. Before the outbreak in Blantyre, we estimated that typhoid fever incidence was 26.1 cases (95% CrI: 14.0–45.7 cases) per 100,000 person-years, which represents a fairly limited health burden, with an average of only 14 culture-confirmed cases per year at QECH [25]. Under these circumstances, we found that no vaccination is the preferred strategy for WTP thresholds less than $900. In general, previous analyses have found that TCV introduction is unlikely to be cost-effective at WTP thresholds below $1000 when incidence is less than 30–50 cases per 100,000 person-years [23, 24, 31]. Similarly, we estimated that the post-outbreak incidence in Blantyre was 224 typhoid fever cases (95% CrI: 169–368 cases) per 100,000 person-years, and routine vaccination with a catch-up campaign was cost-effective at WTP thresholds of $150 and above, similar to results of a previous economic evaluation for Malawi [23]. Considering the likely cost-effectiveness for this current scenario, decision-makers in Malawi chose to introduce TCVs and applied for Gavi support in 2020.

Due to suboptimal surveillance and diagnostics, there is considerable uncertainty surrounding the incidence and burden of typhoid fever, which leads to uncertainty in the preferred vaccination strategy. Nevertheless, some of the uncertainty has been reduced in our analysis compared to previous cost-effectiveness analyses for Malawi. In Bilcke et al. [26], lack of data surrounding the probability of hospitalization, the case fatality risk among inpatients, vaccine delivery costs, and typhoid incidence contributed substantially to uncertainty in the cost-effectiveness of TCV introduction. Since then, additional data have been collected in Malawi [42, 49]. While the parameters contributing most to uncertainty in our analysis included parameters that were and were not updated with new data, the overall expected value of information for the parameters contributing the most to uncertainty was substantially lower compared to the previous cost-effectiveness analyses.

There are several important limitations to our analysis. We assume TCV reduces the burden of typhoid fever by reducing the number of infections and lowering transmission. However, we lack direct data on vaccination impact in this population. We used results from the recent TyVAC trial in Malawi to update our estimates of vaccine efficacy and duration of protection, but observations of vaccine impact following widespread implementation are not yet available. Additionally, the duration of vaccine protection is not fully understood. While we incorporated substantial uncertainty associated with this parameter in our analyses, longer follow-up is needed to validate our assumptions. This analysis also uses parameters and data based on an outbreak in one location. Since the timing of a typhoid fever outbreak is unknown, it is difficult to plan for control. The peak and length of outbreaks, as well as treatment costs and severity of disease, may differ in other contexts. It can also be difficult to collect site-specific data, as typhoid fever surveillance is limited in many countries. We made every effort to incorporate additional uncertainty in the model parameters (which were not only Malawi-specific) and the outbreak itself (using a stochastic model that varied the peak and length of the outbreak). The framework we present may be generalizable to other settings where the introduction of drug-resistant strains may lead to prolonged outbreaks of typhoid fever. However, we do not fully understand why outbreaks occur in some places but not others.

With WHO recommendations for TCV use, an increasing body of evidence supporting TCV efficacy in diverse settings, and studies assessing longer-term efficacy and impact underway, governments are looking to prioritize the allocation of resources to prevent typhoid fever. While for most endemic countries the introduction of TCV into routine immunization programs is the preferred option, studies are needed to compare prevention strategies across different settings, including the use of TCV in response to outbreaks. Findings from this analysis can play an essential role in making the case for vaccination in an outbreak setting to reduce the global burden of typhoid fever. However, typhoid control can be expensive, and the majority of the burden of typhoid fever occurs in low- and middle-income countries. Cost-effectiveness analyses are needed to inform decisions for the optimal allocation of funding. Results from this research can inform policy- and decision-making regarding typhoid prevention and control strategies by providing estimates of which strategies are most cost-effective compared to others under which circumstances.

## Supplementary Information

**Additional file 1: Table S1.** Options for disability weights assigned to different healthcare use groups. Disability weights presented for the two options are for infectious disease, acute, and for the specified level of typhoid episode (mild, moderate, or severe). **Table S2.** Input parameters for transmission model and cost-effectiveness analysis, with distributions. Typhoid incidence and age distribution, mortality, antimicrobial resistance, healthcare use, treatment cost, vaccine-related costs, and disability-adjusted life-year uncertainty distributions and their page location in the text are shown. **Table S3.** Predicted disease and economic burden in the absence of vaccination. The median (95% credible interval) estimates for predicted cases, deaths, DALYs, and treatment costs per 100,000 people over 10 years are shown for each scenario in the absence of vaccination. **Table S4.** Predicted vaccine impact with randomized outbreak timing, pre-outbreak incidence and post-outbreak incidence per 100,000 individuals. The median (95% credible interval) estimates for averted cases, deaths, DALYs, treatment costs, costs of vaccinations and net costs per 100,000 people are shown for each vaccination strategy compared to no vaccination for randomized outbreak timing (“randomized”), pre-outbreak incidence (“pre”), and post-outbreak incidence (“post”). Vaccination strategies include routine vaccination at nine months of age in year 0 with or without a catchup campaign up to 15 years of age. Reactive routine vaccination strategies (with a catchup campaign) include delays of 1, 6, 12, and 24 months to deployment (“1 m”, “6 m”, “12 m”, “24 m”, respectively). All values are presented as incidence per 100,000 people. R = routine vaccination; RC = routine vaccination plus a catchup campaign. **Table S5.** Predicted vaccine impact with fixed outbreak timing incidence per 100,000 individuals. The median (95% credible interval) estimates for averted cases, deaths, DALYs, treatment costs, costs of vaccinations and net costs per 100,000 people are shown for each vaccination strategy compared to no vaccination with fixed outbreak timing. vaccination strategies include routine vaccination at nine months of age in year 0 with or without a catchup campaign up to 15 years of age. Preventive strategy results are shown for 10, 5, 2, and 1 year(s) (“10y”, “5y”, “2y”, “1y”) before the outbreak starts. Reactive routine vaccination strategies (with a catchup campaign) include delays of 1, 6, 12, and 24 months to deployment (“1 m”, “6 m”, “12 m”, “24 m”, respectively). All values are presented as incidence per 100,000 people. R = routine vaccination; RC = routine vaccination plus a catchup campaign. **Table S6.** Predicted vaccine impact with WHO-CHOICE cost of illness data: randomized outbreak timing, pre-outbreak incidence and post-outbreak incidence. The median (95% credible interval) estimates for averted treatment costs and net costs per 100,000 people are shown (averted cases, deaths, DALYs, and costs of vaccinations are the same as the main scenarios) for each vaccination strategy compared to no vaccination for randomized outbreak timing (“randomized”), pre-outbreak incidence (“pre”), and post-outbreak incidence (“post”) using previous WHO-CHOICE cost of illness data. Vaccination strategies include routine vaccination at nine months of age in year 0 with or without a catchup campaign up to 15 years of age. Reactive routine vaccination strategies (with a catchup campaign) include delays of 1, 6, 12, and 24 months to deployment (“1 m”, “6 m”, “12 m”, “24 m”, respectively). All values are presented as costs per 100,000 people. R = routine vaccination; RC = routine vaccination plus a catchup campaign. **Fig S1.** Ordinary differential equations and corresponding dynamic compartmental model for typhoid disease dynamics. Black compartments and text indicate the scenario in which there is no vaccination, and the blue compartments and text indicate the added scenarios in which vaccination is introduced. Note that this model is also age-structured, though not shown. **Fig S2.** Observed and modeled vaccine efficacy over time. Observed data from a phase 3, double-blind, randomized active-controlled clinical trial of single-dose TCV in Blantyre, Malawi at 3 time points (12, 18, and 24 months) (black points with 95% confidence interval error bars) and the modeled vaccine efficacy after vaccination (median estimate: solid blue line; 95% credible interval in shaded light blue) are shown. **Fig S3.** Sensitivity and specificity of outbreak identification threshold definitions. The sensitivity (purple) and specificity (green) are shown for each outbreak identification definition (x-axis; ranging from 6–16 standard deviations above the mean monthly reported typhoid fever cases). **Fig S4.** Estimated specificity of outbreak identification thresholds 6 to 16 standard deviations above the monthly mean reported typhoid fever cases. The median outbreak identification date (dot) and 95% credible interval (line) is shown for outbreak identification thresholds of 6–16 standard deviations above the monthly mean number of typhoid cases for 1000 simulations of the dynamic model. The black dashed line represents the “true” start date of the outbreak, and the shaded grey area represents 0–18 months after the outbreak started (sensitivity window). **Fig S5.** Observed and fitted proportion of typhoid infections that are resistant to antimicrobial treatment in Blantyre, Malawi from 1995–2025. Observed data points of the yearly proportion of antimicrobial resistant typhoid fever infections over time are shown in black dots, while the fitted estimates from the beta regression model are shown in the dashed blue line and the prediction intervals are shown in the turquoise dotted lines. **Fig S6.** Predicted and observed weekly blood-culture confirmed typhoid fever cases in the absence of vaccination. The 1000 stochastic realizations of weekly blood-culture confirmed typhoid fever case incidence per 100,000 people in the absence of vaccination from the dynamic transmission model are show in purple. The observed (reported) typhoid fever cases used to fit the dynamic model is represented by the bold black line, while the observed incidence collected after model fitting is represented by the dashed red line. **Fig S7.** Observed versus fitted age distribution of reported typhoid cases. The proportion of observed cases in each age group are denoted by light blue bars, while the model-predicted age distribution is shown in darker blue. **Fig S8.** Predicted weekly blood-culture confirmed typhoid infections in Scenario 1 for reactive vaccination strategies. The 1000 simulated predictions for weekly blood-culture confirmed typhoid infections are shown in purple, with the median of all stochastic realizations shown in orange for each reactive vaccination strategy. Four situations are shown, representing the four different delays in timing to implement vaccination once the outbreak is identified (1, 6, 12, and 24 months). The median typhoid infections in the absence of vaccination from 1000 realizations is shown in black, and the median date of vaccination deployment for each situation is denoted by the vertical dashed green line. **Fig S9.** Predicted weekly blood-culture confirmed typhoid fever cases in Scenario 1 for preventive vaccination strategies. The 1000 stochastic realizations of weekly typhoid cases are shown in purple, with the median of all simulations shown in orange for each preventive vaccination strategy. Eight situations are shown, representing each preventive routine vaccination timing strategy (10, 5, 2, and 1 year(s) before the outbreak for routine vaccination at 9 months of age with and without a catchup campaign up to 15 years of age). The median number of typhoid infections in the absence of vaccination from 1,000 realizations is shown in black, and the date of vaccination deployment for each situation is denoted by the vertical dashed green line. **Fig S10.** Heatmap of optimal intervention strategy and its estimated uncertainty across a range of willingness to pay values for each strategy comparison and a range of deployment delays, and years before the outbreak. Each column in a single panel shows the preferred strategy (i.e. the strategy that yields the highest average net benefit) for one cost-effectiveness analysis comparing no vaccination (grey), preventive routine vaccination (purple), preventive routine vaccination with a catch-up campaign (green), and reactive vaccination with a catch-up campaign (orange) for delays of 1, 6, 12, or 24 months after the outbreak has been identified (x-axis). The y-axis represents willingness-to-pay (WTP) values ranging from $0-$1000 (USD 2020). The shading represents the probability that the preferred strategy yields the highest net benefit (lighter: lower probability; darker: higher probability). Results are plotted for whether preventive vaccination is introduced 10 years (top panel) or 1 year (bottom panel) before the outbreak for a 20-year time horizon. Note that preventive routine vaccination without a catchup campaign is never a preferred strategy, and as a result does not appear in the plots. **Fig S11.** Cost-effectiveness planes and acceptability frontiers for sensitivity analysis assuming WHO-CHOICE treatment costs. The cost-effectiveness planes (left) and cost-effectiveness acceptability frontiers (CEAFs; right) are plotted for (A-B) Scenario 1 (randomized outbreak timing), (C-D) Scenario 2 (no outbreak, assuming pre-outbreak incidence), and (E–F) Scenario 3 (outbreak has already occurred). In the cost-effectiveness planes, each dot represents the incremental costs (in 2020 USD) and DALYs averted for one simulation when compared with the base case strategy of no vaccination. The bold Xs denote the expected additional cost and DALYs averted for each vaccination strategy with respect to no vaccination. Strategies are indicated by the color of the dot or X (purple: preventive routine vaccination; green: preventive routine vaccination plus a catch-up campaign up to age 15; or orange: reactive routine vaccination plus a catch-up campaign to age 15—for Scenario 1 only). In the CEAFs, the preferred strategy (i.e. the strategy that yielded the highest *average* net benefit) for each willingness-to-pay threshold ($0–1,000 per DALY averted; x-axis, 2020 USD) is indicated by the color of the line (black: no vaccination; and same strategy colors as other panels), while the proportion of samples in which that strategy yielded the highest net benefit is indicated by the value on the y-axis; this can be interpreted as our certainty in the optimal strategy. **Fig S12.** Expected value of partially perfect information for differing delays in vaccination deployment for reactive strategies with randomized outbreak timing (Scenario 1). The expected value of partial perfect information (EVPPI) for each parameter is shown for a range of willingness-to-pay values. Results are shown for 5000 parameter samples (in 2020 USD). Each panel shown represents the EVPPI for one cost-effectiveness analysis comparing 4 strategies: no vaccination (base case), preventive routine vaccination at 9 months, preventive routine vaccination with a catch-up campaign up to 15 years, and reactive routine vaccination with a catch-up campaign. The four panels (left to right) correspond to different delays in the reactive strategy (1-, 6-, 12-, or 24-month delays). The grey vertical dashed line corresponds to the 2020 gross domestic product per capita for Malawi. **Fig S13.** Expected value of partially perfect information for Scenario 2 (pre-outbreak incidence). The expected value of partial perfect information (EVPPI) for each parameter is shown for a range of willingness-to-pay values. Results are shown for 5000 parameter samples in 2020 USD. **Fig S14.** Expected value of partially perfect information for Scenario 3 (post-outbreak incidence). The expected value of partial perfect information (EVPPI) for each parameter is shown for a range of willingness-to-pay values. Results are shown for 5000 parameter samples in 2020 USD.

## Data Availability

The data and code used for the dynamic model are available at https://github.com/mailephillips/typhoid-outbreak_Malawi.
