# Supplementary material for: Cost-effectiveness analysis of typhoid conjugate vaccines in an outbreak setting: a modeling study
Source: BMC Infect Dis. 2023 Mar 8;23:143. doi: 10.1186/s12879-023-08105-2 (PMC9993384; doi:10.1186/s12879-023-08105-2)
Supplement: Supplementary file 1 — Additional file 1: Table S1. Options for disability weights assigned to different healthcare use groups. Disability weights presented for the two options are for infectious disease, acute, and for the specified level of typhoid episode (mild, moderate, or severe). Table S2. Input parameters for transmission model and cost-effectiveness analysis, with distributions. Typhoid incidence and age distribution, mortality, antimicrobial resistance, healthcare use, treatment cost, vaccine-related costs, and disability-adjusted life-year uncertainty distributions and their page location in the text are shown. Table S3. Predicted disease and economic burden in the absence of vaccination. The median (95% credible interval) estimates for predicted cases, deaths, DALYs, and treatment costs per 100,000 people over 10 years are shown for each scenario in the absence of vaccination. Table S4. Predicted vaccine impact with randomized outbreak timing, pre-outbreak incidence and post-outbreak incidence per 100,000 individuals. The median (95% credible interval) estimates for averted cases, deaths, DALYs, treatment costs, costs of vaccinations and net costs per 100,000 people are shown for each vaccination strategy compared to no vaccination for randomized outbreak timing (“randomized”), pre-outbreak incidence (“pre”), and post-outbreak incidence (“post”). Vaccination strategies include routine vaccination at nine months of age in year 0 with or without a catchup campaign up to 15 years of age. Reactive routine vaccination strategies (with a catchup campaign) include delays of 1, 6, 12, and 24 months to deployment (“1 m”, “6 m”, “12 m”, “24 m”, respectively). All values are presented as incidence per 100,000 people. R = routine vaccination; RC = routine vaccination plus a catchup campaign. Table S5. Predicted vaccine impact with fixed outbreak timing incidence per 100,000 individuals. The median (95% credible interval) estimates for averted cases, deaths, DALYs, treatment costs, costs o [file 12879_2023_8105_MOESM1_ESM.docx]

**Additional file: *Cost-effectiveness analysis of typhoid conjugate vaccines in an outbreak setting***

**Authors:** Maile T. Phillips^1^, Marina Antillon^2^, Joke Bilcke^4^, Naor Bar-Zeev^5,6^, Fumbani Limani^6^, Frédéric Debellut^7^, Clint Pecenka^8^, Kathleen M. Neuzil^9^, Melita A. Gordon^6,10^, Deus Thindwa^6,11^, A. David Paltiel^12^, Reza Yaesoubi^12^, Virginia E. Pitzer^1^

**Author affiliations:**

1. Department of Epidemiology of Microbial Diseases, Yale School of Public Health, New Haven, Connecticut, United States of America
2. Swiss Tropical and Public Health Institute, Basel, Switzerland
3. University of Basel, Basel, Switzerland
4. Center for Health Economics Research and Modeling Infectious Diseases, University of Antwerp, Antwerp, Belgium
5. International Vaccine Access Center, Department of International Health, Bloomberg School of Public Health, Johns Hopkins University, Baltimore, Maryland, United States of America
6. Malawi Liverpool Wellcome Programme, Kamuzu University of Health Sciences, Blantyre, Malawi
7. Center for Vaccine Innovation and Access, PATH, Geneva, Switzerland
8. Center for Vaccine Innovation and Access, PATH, Seattle, Washington, United States of America
9. Center for Vaccine Development, University of Maryland School of Medicine, Baltimore, MD, United States of America
10. Institute of Infection, Veterinary and Ecological Sciences, University of Liverpool, Liverpool, United Kingdom
11. Department of Infectious Disease Epidemiology, London School of Hygiene and Tropical Medicine, London, United Kingdom
12. Department of Health Policy, Yale School of Public Health, New Haven, Connecticut, United States of America

**S1 Additional Methods**

***S1.1.* *Transmission-dynamic model***

***S1.1.1****. Description of the dynamic model.* We simulated the conditions under which an outbreak may occur using an existing dynamic transmission model. Briefly, in this model, individuals are born completely susceptible to typhoid, and they move through susceptible, infectious, chronic carrier, and immune compartments as specified through a system of differential equations (deterministic model) or probabilistic transitions (stochastic model) (Fig S1). Individuals in the susceptible population (*S_1_*) become infected at rate $\lambda$. Primary infections (*I_s_*) may be symptomatic and remain infectious for length of time $\frac{1}{\delta}$, after which they experience one of three options: a proportion die from typhoid ($\alpha)$, a fraction $\theta_{a}$ become chronic carriers ($C$), and the remaining $1-\theta_{a}-\alpha$ recover and are temporarily immune ($R$). Since age (*a*) is a factor in the development of the chronic carrier state [1], $\theta_{a}$ is age-dependent. Immune individuals lose immunity and become susceptible to reinfection ($S_{2}$) at rate $\omega$. If an individual becomes re-infected, we assume the infection is subclinical ($I_{A}$). Subclinically infected people can recover (at rate $\delta(1-\theta_{a})$). The same process of reinfection can occur. In all compartments, individuals die from non-typhoid causes at rate $\mu_{a}$ and age into the next age group at a rate inversely proportional to the width of the age group.

Symptomatic and subclinically infected individuals and chronic carriers all contribute to the force of infection, although chronic carriers contribute at a reduced rate ($r)$. The force of infection $\lambda_{a}$ is age-dependent (where *a =* 0 to <9 months; 9 months to <5 years; five-year-interval age groups from 5 to <80 years; and 80 years and older) and is the product of the age-dependent transmission rate ($\beta_{a}$) and the sum of all infectious states, divided by the total population *N*: $\lambda_{a}= \frac{\beta_{a}}{N_{a}}\sum_{\text{All ages}} {(I}_{S,a}+I_{A,a}+rC_{a})$.


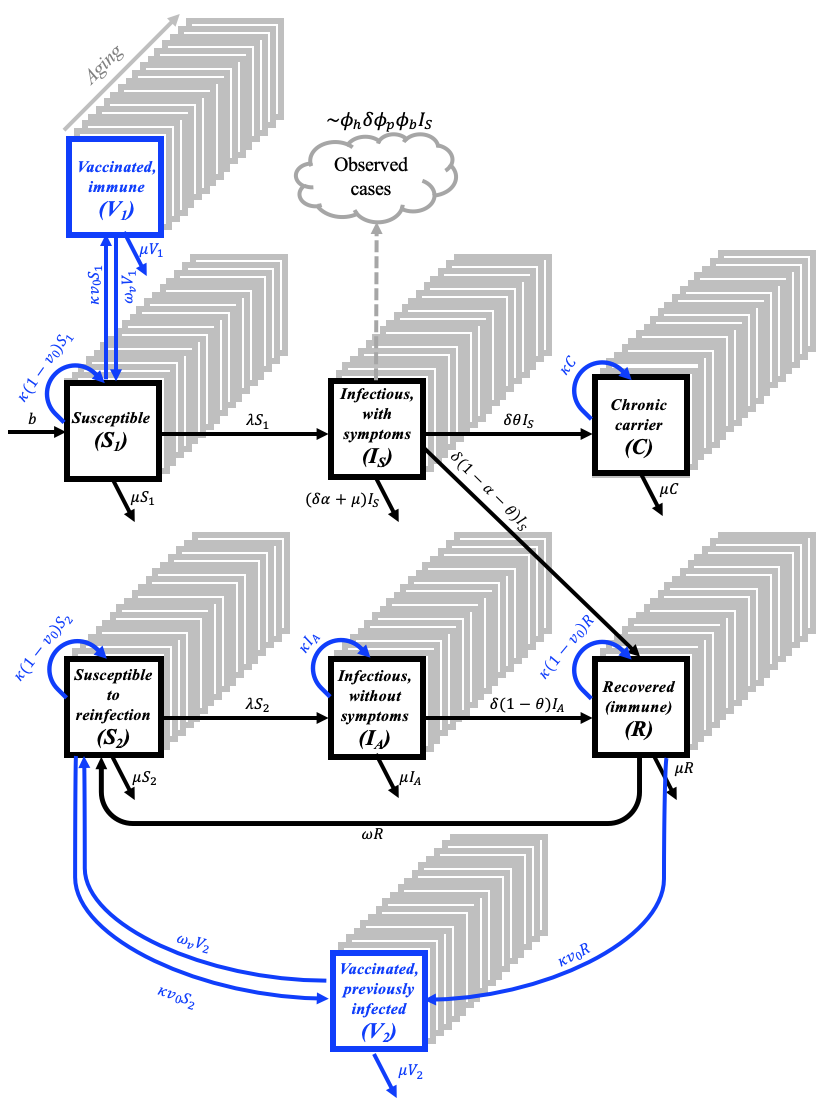


With and without vaccination

$$\frac{dS_{1}}{dt}=bN-\lambda S_{1}-\mu S_{1}-\kappa\nu_{0}S_{1}+\omega_{v}V_{1}s$$

$$\frac{dI_{S}}{dt}=\lambda S_{1}-\delta I_{S}-\mu I_{S}$$

$$\frac{dR}{dt}=\delta\left( 1-\theta-\alpha\right)I_{S}+\delta\left( 1-\theta\right)I_{A}-(\omega+\mu)R-\kappa\nu_{0}R$$

$$\frac{dC}{dt}=\delta\theta I_{S}-\mu C$$

$$\frac{dS_{2}}{dt}=\omega R-\lambda S_{2}-\mu S_{2}-\kappa\nu_{0}S_{2}$$

$$\frac{dI_{A}}{dt}=\lambda S_{2}-\delta I_{A}-\mu I_{A}$$

$$\frac{dV_{1}}{dt}=\kappa\nu_{0}S_{1}- \omega_{V}V_{1}-\mu V_{1}$$

$$\frac{dV_{2}}{dt}=\kappa\nu_{0}(S_{2}+R)- \omega_{V}V_{2}-\mu V_{2}$$

**Fig S1. Ordinary differential equations and corresponding dynamic compartmental model for typhoid disease dynamics.** Black compartments and text indicate the scenario in which there is no vaccination, and the blue compartments and text indicate the added scenarios in which vaccination is introduced. Note that this model is also age-structured, as depicted by the grey boxes. We assume exponential aging at a rate equal to the width of the age group.

Observed symptomatic cases are a fraction $\phi^{S}\phi^{B}\phi^{H}$ of the true number of cases (S1.1.4 Text) [2]. The culture-confirmed cases are adjusted to account for the sensitivity of the blood culture test ($\phi^{S}$), the probability of receiving a blood culture test ($\phi^{B}$), and the probability of healthcare seeking ($\phi^{H}$), which occurs sometime during the period $\frac{1}{\delta}$ of infection.

To adjust for the outbreak and vaccination scenarios, we simulated the weekly number of symptomatic cases for the 18 age groups (0 to <9 months; 9 months to <5 years; five-year-interval age groups from 5 to <80 years; and 80 years and older). We modelled the emergence of multidrug-resistant typhoid by allowing for an increase in the duration of infectiousness. We estimated the date it began to increase (April 10, 2011) and the date it stabilized at a new higher value (November 23, 2014), which is consistent with the timing of the emergence of multi-drug resistance in Blantyre [3]. The magnitude of the increase *m* was estimated to be 3.1954. For further details, see Pitzer et al [4].

To stay consistent with recent estimates of the contribution of chronic carriers to transmission, which is associated with the level of indirect protection from vaccination, we applied a prior distribution of $Beta(6.34,19.4)$ on the range of values for *r* in the model-fitting process [5]. With these changes, we re-fit the dynamic model using the same process to the same Malawi outbreak data to update the remaining parameters. We estimated the pre-outbreak reproductive number (*R_0_*), the amplitude of seasonal forcing (*q*), the relative infectiousness of chronic carriers (*r*), the beginning and ending week of the increase in duration of infectiousness during the outbreak (*t_1_* and *t_2_*), and the magnitude of that increase in the duration of infectiousness (*m*) by fitting the compartmental model to the weekly number and age distribution of culture-confirmed cases of typhoid fever at Queen Elizabeth Central Hospital in Blantyre between January 1996 and February 2015 using maximum likelihood methods. Weekly cases were assumed to be Poisson-distributed, while the age distribution was assumed to follow a multinomial distribution. This dynamic model and the model-fitting process are described in more detail elsewhere [4].

***S1.1.2****. Modeling vaccination.*

***S1.1.2.1.*** *Vaccination compartments.* To simulate vaccination and measure the overall impact of each strategy (with vaccine coverage *κ*), we added additional vaccination compartments to the dynamic model (Fig S1, in blue). We assumed that individuals who successfully mount a protective immunological response to the vaccine are moved into these compartments and are protected from infection and disease while vaccine-induced immunity lasts. While in these vaccinated compartments, individuals are also removed from the transmission cycle. This assumption is based on evidence from human challenge studies that TCVs also reduced stool shedding of *S.* Typhi [6], as well as evidence from cluster randomized trials that Vi-polysaccharide vaccines conferred indirect protection when high population-level coverage was achieved [7].

We added two vaccination compartments to the transmission model. One compartment (*V_1_*) represents individuals who have never been infected and hence may be protected from symptomatic disease if they successfully mount a protective immunological response to the vaccine. The second compartment (*V_2_*) represents those who were previously infected and would only be protected against reinfection (and hence transmission), since they already have immunity to clinical disease. In both cases, vaccine-induced immunity eventually wanes at rate $\omega_{V}$ (S1.1.2.2).

***S1.1.2.2.*** Vaccine efficacy and waning of vaccine-induced immunity

Typbar TCV (Bharat Biotech International) is the first WHO-pre-qualified typhoid conjugate vaccine (TCV). This TCV was licensed based on improved immunogenicity data (compared to previous typhoid fever vaccines) and efficacy data from a human challenge study [8-10]. Randomized controlled trials of Typbar TCV have recently been completed in Nepal, Bangladesh, and Malawi and have shown the vaccine to be efficacious [11-13]. For this analysis, we used the results for 24 months of follow-up from the phase 3, double-blind, randomized active-controlled clinical trial of single-dose TCV in Blantyre, Malawi [11, 14, 15]. These are the first vaccine efficacy and safety results from Africa.

The TCV trial in Blantyre provided observed values for vaccine efficacy at 12 months, 18 months, and 24 months of follow-up. To account for waning efficacy over time, we assumed that the mean vaccine efficacy $\nu$ at time *t* followed an exponential decay pattern:

$\nu\left( t \right)=\nu_{0}*e^{-\left( \omega_{V}*t \right)}$,

where $\nu_{0}$ is the initial vaccine efficacy at time 0 (modeled as an all-or-nothing vaccine “take” in the transmission model) and $\omega_{V}$ is the rate of waning of vaccine-induced immunity. The observed response variable vaccine efficacy at time *t* (*VE*(*t*)) was assumed to be normally distributed with mean$\nu(t)$ and standard deviation $\sigma$:

$VE\left( t \right)\sim N\left( \nu\left( t \right), \sigma\right)$.

We used an informative prior on the duration of vaccine-induced immunity, based on data from 4 years of follow-up for the Vi-rEPA TCV: $\frac{1}{\omega_{V}}\sim Gamma\left( 1.38,0.048 \right)$ [5]. We used a noninformative uniform prior on the standard deviation of the normally distributed sampling error of vaccine efficacy at time *t.* These distributions resulted in an estimated initial TCV efficacy of 0.89 (95% CrI: 0.78-0.98) and an estimated duration of vaccine-induced immunity of 18.87 (95% CrI: 8.40-83.33) years. The observed and model-predicted vaccine efficacy at time *t* are shown in Fig S2.

***
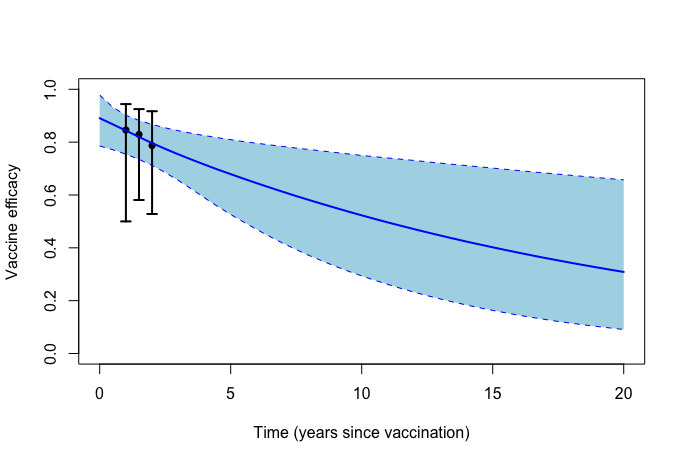
***

**Fig S2. Observed and modeled vaccine efficacy over time.** Observed data from a phase 3, double-blind, randomized active-controlled clinical trial of single-dose TCV in Blantyre, Malawi at 3 time points (12, 18, and 24 months) (black points with 95% confidence interval error bars) and the modeled vaccine efficacy after vaccination (median estimate: solid blue line; 95% credible interval in shaded light blue) are shown.

***S1.1.3****. Outbreak threshold definitions.* We explored a range of thresholds to identify the start of the outbreak. For the main analysis, we varied the number of standard deviations (6-16) above the monthly mean reported typhoid fever cases in the previous 10 years to define the outbreak start. We defined the sensitivity of each threshold as the percentage of simulations in which the outbreak was identified within 18 months of April 2011, while the specificity was defined as the percentage of simulations in which the outbreak threshold was not exceeded prior to April 2011.

*
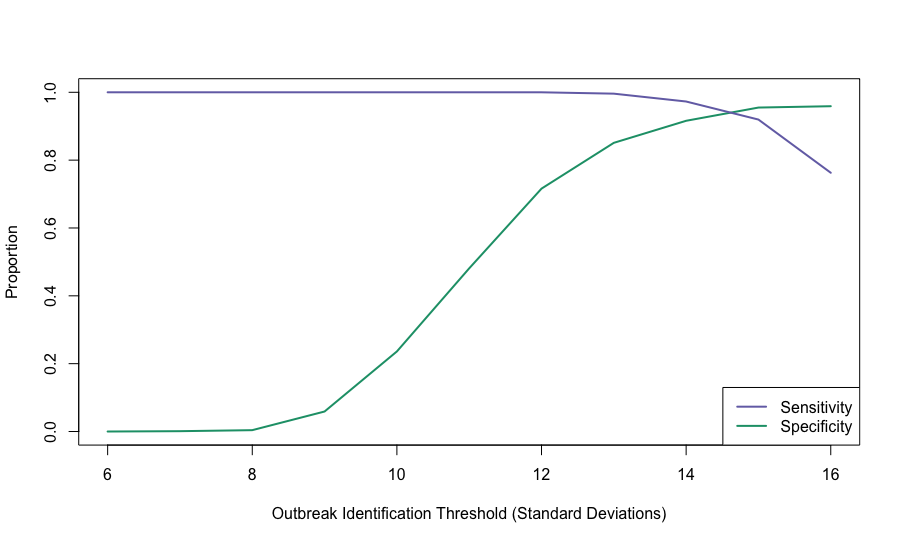
*

**Fig S3. Sensitivity and specificity of outbreak identification threshold definitions.** The sensitivity (purple) and specificity (green) are shown for each outbreak identification definition (x-axis; ranging from 6-16 standard deviations above the mean monthly reported typhoid fever cases).

Across the range of different outbreak identification thresholds we explored, the sensitivity remained high (76.3-100.0%), while the specificity followed a sigmoid pattern (Fig S3). At a threshold of 16 standard deviations above the monthly mean number reported cases, the outbreak was identified within one and a half years of the pre-specified start date with a specificity of 96.5% and a sensitivity of 76.3% (out of 1,000 stochastic iterations); a threshold of 15 standard deviations identified the outbreak with a specificity 95.5% and sensitivity of 91.9%; while a threshold of 12 standard deviations identified it with a specificity of 71.6% and sensitivity of 100%. Lower thresholds exhibited poor specificity and incorrectly identified the outbreak as occurring before April 2011 more than 50% of the time (Fig S4).


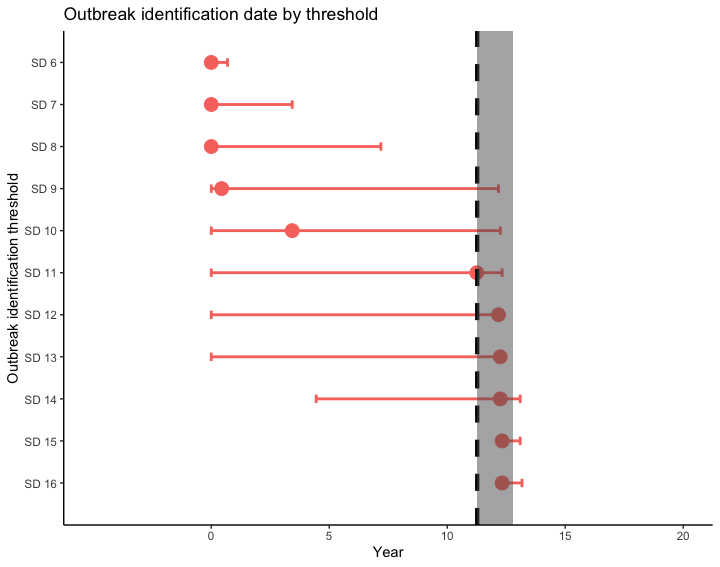


**Fig S4.** **Estimated specificity of outbreak identification thresholds 6 to 16 standard deviations above the monthly mean reported typhoid fever cases.** The median outbreak identification date (dot) and 95% credible interval (line) is shown for outbreak identification thresholds of 6-16 standard deviations above the monthly mean number of typhoid cases for 1,000 simulations of the dynamic model. The black dashed line represents the “true” start date of the outbreak, and the shaded grey area represents 0-18 months after the outbreak started (sensitivity window).

We also explored other outbreak threshold definitions. We defined outbreaks by raw counts of blood-culture confirmed cases and incidence rates, but these definitions, while reliable for identifying the outbreak, would be less applicable across countries because of the variation in incidence and population denominators for passive hospital-based surveillance across settings. We also tried to account for seasonal variation in typhoid fever incidence by allowing each month of the year to have a standard deviation threshold above the mean; however, while this method accounted for seasonality, the data were sparse and oftentimes the outbreak was never identified. We did not want to include more data (farther back than 10 years) because many typhoid-endemic countries do not have long-established typhoid fever surveillance platforms.

***S1.1.4****.* Underreporting adjustment. To adjust for underreporting of observed typhoid cases, we used estimates from an analysis of data from the STRATAA study, which examined the population-based incidence of typhoid fever in Malawi, Nepal, and Bangladesh [2]. We based our estimate of the adjustment factor for Malawi on the posterior distribution obtained by adjusting for blood culture sensitivity, the probability of receiving a blood culture diagnostic test, and healthcare seeking. In Malawi, this adjustment factor was estimated to be 7.7 (95% CrI: 6.0-12.4), i.e. for every blood-culture confirmed case of typhoid fever presenting to healthcare facilities in Blantyre, there are an additional 6.7 undiagnosed cases of symptomatic typhoid fever occurring in the community. This underreporting adjustment was applied after the model was fitted to the reported (unadjusted) number of blood-culture confirmed cases.

***S1.1.5****.* Chronic Carriers. We explored the contribution of chronic carriers to transmission during the model-fitting process. In previous model-fitting, the parameter for the relative infectiousness of chronic carriers was unidentifiable. There is a tradeoff between this parameter and the reproductive number *R_0_*, which influences the prevalence of chronic carriers in the population. To explore this tradeoff, we fixed the relative infectiousness of chronic carriers *r* at different values (0.01, 0.10, 0.25, and 0.50) and refit the remaining parameters. We also tried a variation of model-fitting where we initialized the model with no chronic carriers prior to the 46-year burn-in period.

The overall model fit (as measured by Akaike Information Criterion) tended to be poorer as *r* got farther away from the 0.1-0.5 range. This range is consistent with recent estimates of *r* (based on fitting to the observed overall and indirect effectiveness of vaccination with Vi-polysaccharide vaccine in a cluster-randomized trial in Kolkata [16, 17]) and what was used in the final model. To stay consistent with recent estimates of the contribution of chronic carriers to transmission, we applied a prior distribution of $Beta(6.34,19.4)$ on the range of values for *r* in the model-fitting process [5]. The mean of this prior distribution (0.25) is also within the range of our findings in the sensitivity analysis of the contribution of chronic carriers to transmission.

***S1.2.* Input parameters for economic model**

***S1.2.1.*** *Cost conversion and inflation*

All costs were converted to 2020 USD. First, they were converted from their original amount to USD in the same reported year using WHO exchange rates [18]. Second, they were inflated to the year 2020 using the consumer price index [19].

***S1.2.3.*** *Vaccination costs*

In our analysis, we included only the costs to be paid by the government of Malawi (i.e. accounting for Gavi support). Malawi is currently considered to be in the initial self-financing phase of Gavi support, which means its contribution is 13% of the price per dose for routine vaccination and 0% of the price for campaign doses. Thus, we assumed a vaccine procurement cost of $0.20 per routine dose and $0 for campaign doses ($1.50/dose prior to Gavi support). We also accounted for Gavi support for the delivery of routine doses in the first year of vaccination ($0.80/dose). For routine doses in Malawi, this delivery cost was assumed to be $1.61 (95% confidence interval (CrI): $0.36-4.23) per dose based on the price of adding a new vaccine to routine vaccination across Gavi-eligible countries [5]; for campaign doses, this cost was $0.40 (95% CrI: $0.23-0.62) based on a literature review that explored operational costs per vaccine doses for Supplementary Immunization Activities from 1992-2012 [20]. We also assumed the price to be $0.23 (95% CrI: $0.21-0.24) per dose for injection and safety equipment, as in a previous study [5].

***S1.2.2.*** *Treatment costs*

Direct medical cost estimates with uncertainty for treating inpatients and outpatients for typhoid fever from the healthcare provider’s perspective were available for Blantyre, Malawi in 2020 USD [21]. From Limani et al, we used treatment costs for all ages, which included drugs and medical supplies, laboratory investigations, and bed-day (food, laundry, electricity, water, and security; inpatient only) costs for inpatients ($214.38; 95% CI: $164.41-$264.34) and outpatients ($39.67; 95% CI: $33.96-$45.39). For the treatment costs for individuals with typhoid fever who did not seek formal medical care, we used the cost of drugs for outpatients in the same population as a proxy ($1.59, 95% CI: $0.31-$2.86) [21].

As a scenario analysis, we also conducted the analysis using previous global (not Malawi-specific) treatment costs from WHO-CHOICE data: inpatient ($34.00; 95% CI: $9.00-107.00), outpatient ($1.30; 95% CI: $0.30-3.20), and non-medical ($0.81; 95% CI: $0.039-2.28) [5]. See Bilcke et al [5] for details.

***S1.2.3.*** *Disability weights*

We used mild, moderate, and severe disability weights for acute infectious diseases from the 2010 Global Burden of Disease study to reflect the severity of typhoid fever compared to other diseases [22, 23]. We chose to use the 2010 study instead of the newer estimates due to the fact that newer disability weights are based on updated data from upper-income countries that do not reflect the populations in which typhoid fever is endemic. Disability weights for mild episodes were characterized as having “low fever and mild discomfort, but no difficulty with daily activities,” estimated to be $0.005\pm0.002$ [23]; for moderate episodes they were characterized as having “a fever and aches and feels weak, which causes some difficulty with daily activities,” estimated to be $0.053\pm0.012$ [23]; and for severe episodes they were characterized as having “a high fever and pain, and feels very weak, which causes great difficulty with daily activities,” estimated to be $0.210\pm0.040$ [23]. These mild, moderate, and severe categorizations corresponded to inpatient, outpatient, and non-medical care episodes of typhoid fever in two different ways, resulting in different average disability weights, each with equal probability of occurring (Table S1).

We assumed the duration of illness increases with the need for medical care. In this case, we used the distribution of duration of illness for inpatients and outpatients from a previous random effects model by Bilcke et al. that integrated multiple estimates [5]. Similar to the previous study, we also assumed that individuals with typhoid who did not seek medical care recovered from illness twice as fast as those who sought care.

**Table S1.** **Options for disability weights assigned to different healthcare use groups.** Disability weights presented for the two options are for infectious disease, acute, and for the specified level of typhoid episode (mild, moderate, or severe).

|  | **Option 1** | **Option 2** |
| --- | --- | --- |
| *Inpatient* | Severe | Severe |
| *Outpatient* | Moderate | Severe |
| *Patient not seeking medical care* | Mild | Moderate |
| ***Average disability weight*** | **0.04** | **0.15** |

***S1.2.4.*** *Probability of seeking professional medical care*

To calculate the probability of seeking medical care for typhoid fever, we again used estimates from the STRATAA study [2]. As part of STRATAA, healthcare utilization surveys were conducted in the Ndirande township of Blantyre to assess the probability of seeking care for fever [24]. Many methods assume that reported healthcare-seeking for a fever is the same as that for typhoid fever; however, this is not necessarily the case. Individuals with typhoid fever may be more or less likely to seek healthcare. To correct for this difference, we measured the probability of seeking care for a fever adjusted for a specified typhoid risk factor to estimate the probability of seeking care for typhoid fever. In Malawi, this risk factor was soap available after defecation. After adjusting for this risk factor, the probability of seeking professional medical care in Malawi was approximately 0.71 (95% CrI: 0.64-0.77) [2].

***S1.2.5.*** *Probability of hospitalization*

During the STRATAA study, we observed 8 hospitalizations among 105 blood-culture-confirmed typhoid infections. However, since the focus of this analysis is more broadly on Malawi as an example of a multi-year outbreak of typhoid fever associated with the emergence of AMR, we used the STRATAA data from Blantyre, Malawi to update a previous estimate of the probability of hospitalization among Gavi-eligible countries based on a meta-analysis by Abboud et al [5, 25].

We modeled probability of hospitalization using a binomial distribution for the likelihood contribution, where *y_hosp_* was the observed number of hospitalizations, *p_hosp_* was the probability of hospitalization among culture-confirmed typhoid infections, and *n_hosp_* was the number of typhoid fever cases,

$y_{hosp}\sim Binomial(p_{hosp},n_{hosp})$.

In the previous analysis, the denominator (blood-culture-confirmed infections) was adjusted for differences in surveillance method according to the probability of seeking professional medical care. Using the same model for adjustment as Section 1.1.4, our denominator for the total number of typhoid fever cases in the community could be as large as 429 (median estimate after adjusting for the probability of receiving a blood culture test and the probability of seeking healthcare). However, it is also possible that individuals who do not seek care are less severe and unlikely to be hospitalized. Hence, we allowed the denominator $n_{hosp}$ to vary between 105 and 429 by giving it a discrete uniform prior distribution:

$n_{hosp}\sim Uniform(105,429)$.

The previous meta-analysis estimated the probability of hospitalization to be 0.04 (95% prediction interval (PI): 0.00-0.25). We incorporated this estimate as a prior distribution on the probability of hospitalization using an inverse logit of a normal distribution with mean equal to the common estimate from the Abboud et al meta-analysis ($\mu_{hosp}=-3.25$ on the logit scale), and standard error equal to the standard error based on the prediction interval around the common estimate ($\sigma_{hosp}=1.20$ on the logit scale) [25]:

$$logit\left( p_{hosp} \right)\sim N\left( \mu_{hosp},\sigma_{hosp} \right).$$

Combining the likelihood of the data with the probability of the prior distribution resulted in an estimated probability of hospitalization of 0.04 (95% PI: 0.01-0.11).

***S1.2.6.*** *Probability of death if patients are admitted to hospital for typhoid infection*

In STRATAA, we observed 1 death among the 8 individuals hospitalized for typhoid fever in Malawi, and 1 death occurred in an individual who was not hospitalized. Similar to the probability of hospitalization, we combined the data with prior information from a previous meta-analysis to estimate the case fatality risk (CFR) among inpatients admitted for typhoid fever. We again modeled this estimate using a binomial distribution for the likelihood contribution, where *y_IP.CFR_=*1 was the observed number of deaths among inpatients, *p_IP.CFR_* was the probability of death among inpatients, and *n_IP.CFR_=*8 was the number of hospitalized cases,

$y_{IP.CFR}\sim Binomial\left( p_{IP.CFR},n_{IP.CFR} \right)$.

In this case, we wanted to update our prior to include not only the meta-analysis by Pieters et al [26] used in the previous analysis by Bilcke et al [3], but to also include estimates from a more recent meta-analysis that had been carried out for inpatient CFR estimates by Crump et al [27]. The original inpatient CFR from Pieters et al was estimated to be 0.04 (95% PI: 0.01-0.20) based on 21 studies. Crump et al’s new meta-analysis had a lower pooled inpatient CFR estimate of 0.02 (95% PI: 0.01-0.03) overall (109 studies), or 0.05 (95% PI: 0.03-0.09) for studies based in Africa [27]. To supplement the meta-analysis by Pieters et al, we added studies from Crump et al’s meta-analysis that had the same inclusion criteria (no population subgroups, hospital-based studies only). This exclusion process identified 26 additional studies from Crump et al, 22 of which we were able to locate. Since multi-year typhoid outbreaks are primarily occurring in sub-Saharan Africa, we further limited the studies to only include those in sub-Saharan Africa. With this additional restriction, we included 12 additional studies and the estimated inpatient CFR was 0.07 (95% PI: 0.01-0.38).

We incorporated the updated estimate as a prior distribution using an inverse logit of a normal distribution with mean equal to the common estimate from the updated random-effects meta-analysis in sub-Saharan Africa ($\mu_{IP.CFR}=-2.52)$, and standard error equal to the standard error based on the prediction interval around the common estimate ($\sigma_{IP.CFR}=1.04$; both on the logit scale):

$$logit\left( p_{IP.CFR} \right)\sim N\left( \mu_{IP.CFR},\sigma_{IP.CFR} \right).$$

Combining the likelihood of the data with the probability of the prior distribution resulted in a final estimated inpatient CFR of 0.09 (95% PI: 0.02-0.28).

***S1.2.7.*** *Time-varying proportion of typhoid infections that are resistant to antimicrobial treatment*

Since the recent typhoid outbreaks are thought to be caused by antimicrobial resistant (AMR) strains, the proportion of typhoid infections resistant to antimicrobial treatment is assumed to be time-varying. A recent study from Feasey et al documented the annual proportion of AMR infections at Queen Elizabeth Central Hospital in Blantyre from 1998-2014 [3]. We supplemented these data points with estimates from Blantyre in the community-based STRATAA Programme in 2017-2018. With change point analysis, we identified that the AMR increase began in 2010, consistent with when we estimated the outbreak to begin using our transmission model. We fit a beta regression model with the logit-transformed proportion of AMR infections as the outcome and third-degree polynomial terms of time (year) as the independent variables. The beta regression model ensured that the proportions of AMR stayed in the interval (0,1). We compared commonly used links for the outcome variable (identity, log, log-log, and logit) and differing degrees of polynomial terms for time and chose the model with the lowest Akaike Information Criteria (AIC) value. This model provided time-varying estimates with prediction intervals for 1995-2018. We assumed that after 2018, the proportion AMR stayed the same; we doubled the standard deviations for the prediction intervals to allow for additional uncertainty in our future extrapolation (Fig S5).


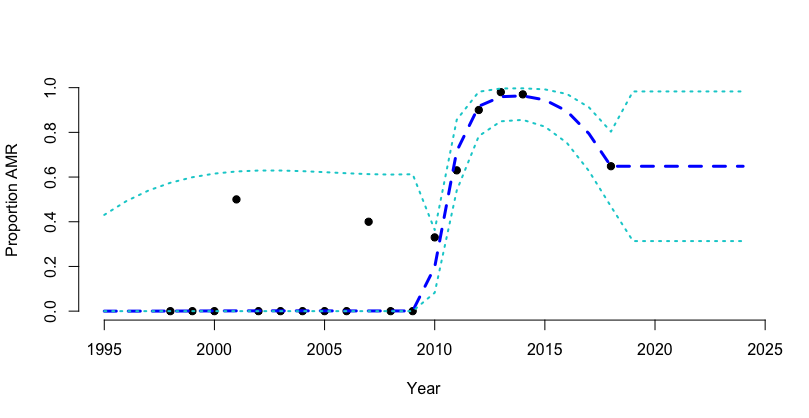


**Fig S5. Observed and fitted proportion of typhoid infections that are resistant to antimicrobial treatment in Blantyre, Malawi from 1995-2025.** Observed data points of the yearly proportion of antimicrobial resistant typhoid fever infections over time are shown in black dots, while the fitted estimates from the beta regression model are shown in the dashed blue line and the prediction intervals are shown in the turquoise dotted lines.

***S1.2.8.*** *All other parameters*

All other parameters not mentioned above that were used in the cost-effectiveness analysis (Table 3) have been described in detail elsewhere [5].

**Table S2. Input parameters for transmission model and cost-effectiveness analysis, with distributions.**

| **Characteristic** | **Uncertainty distribution** | **Page location** |
| --- | --- | --- |
| Typhoid incidence and age distribution | | |
| Annual number of symptomatic typhoid fever cases per 100,000 people (without vaccination) | Estimated from dynamic transmission model | Based on output from transmission dynamic model fit to incidence of typhoid |
| Average age of patients with typhoid infection (without vaccination) (years) | Estimated from dynamic transmission model | Based on output from transmission dynamic model fit to incidence of typhoid |
| Typhoid mortality | | |
| Probability of death if patients are admitted to hospital for typhoid infection | Binomial likelihood (1 success, 8 trials), inverse-logit-normal prior (mean=-2.52; standard deviation=1.04) | Page 11 |
| Proportion of deaths from typhoid infection occurring in patients not hospitalized | Uniform from 0.25 to 1 | [5] |
| Average age at death from typhoid infection | Estimated from dynamic transmission model | Assuming age distribution of deaths is the same as the age distribution of patients with typhoid |
| Antimicrobial resistance | | |
| Proportion of patients with typhoid infection with an AMR strain | Change point analysis; beta regression | [3, 24] |
| Burden of AMR cases relative to antimicrobial-sensitive cases | Uniform from 1 to 3 | [5] |
| Healthcare use | | |
| Probability of infected patients seeking healthcare | Based on posterior distribution obtained by adjusting population-based incidence of typhoid | [2]; page 9 |
| Probability that infected patients are admitted to hospital | Binomial likelihood (8 successes, number of trials follow uniform distribution from 104 to 429), inverse-logit-normal prior (mean=-3.25; standard deviation=1.20) | Page 10 |
| Length of stay in hospital (days) | Gamma distribution based on meta-analysis | [5] |
| Treatment, vaccine-related costs | | |
| All treatment and vaccine-related costs | Gamma distribution based on cost of illness estimates | [21]; page 9 |
| Disability-adjusted life-years | | |
| Disability-weights from 0 (perfect health) to 1 (death) | Equal probability based on two scenarios (Table S1) | [23]; page 8 |
| Duration of illness in inpatients and outpatients (days) | Based on meta-analyses | [5] |
| Relative duration of illness for patients not seeking medical care (vs inpatients and outpatients) | Uniform from 0 to 1 | [5] |
| Life expectancy | Fixed | [28] |

***S1.3.* Cost-effectiveness analysis.** *Determining the optimal strategy for each scenario.*

We calculated the associated costs and disability-adjusted life-years (DALYs) for each strategy (preventive routine vaccination, preventive routine vaccination with a catchup campaign, and reactive routine vaccination with a catchup campaign) and compared to no vaccination using the net monetary benefit (NMB) framework, where $NMB=\Delta E*WTP-\Delta C$ (where $\Delta E$ is the averted DALYs through a vaccination strategy compared to the base case of no vaccination, $\Delta C$ is the incremental cost of the vaccination strategy compared to the base case, and *WTP* is the willingness-to-pay threshold). Our measure of cost-effectiveness is the incremental net monetary benefit as opposed to the incremental cost-effectiveness ratio (ICER), because calculating the preferred strategy for more than two strategies is not always straightforward when using ICERs.

Since there is no standard WTP threshold to define whether an intervention is “cost-effective,” we identified the optimal strategy (i.e. the strategy with the highest average NMB) for WTP values ranging from $0-$1,000 per DALY averted. For comparison, Malawi’s 2019 gross domestic product (GDP) per capita was $411.55 [29], within the range of these WTP values.

***S1.3.1.*** *Uncertainty surrounding the optimal strategy in each analysis*

We randomly drew 5,000 independent samples from the uncertainty distributions of each input parameter in the economic evaluation and combined each sample with one of the samples from the stochastic transmission model to estimate 5,000 net monetary benefit values for each strategy and for a range of WTP values from $0-$1,000 in increments of $10 per DALY averted. The proportion of the 5,000 samples for which the strategy with the higher *average* NMB also has the highest NMB among all strategies for that particular sample reflects our certainty regarding the preferred strategy, presented as a cost-effectiveness acceptability frontier (CEAF). For example, in Scenario 2 (pre-outbreak), the preferred strategy is no vaccination for all WTP values less than $900. However, the probability that no vaccination results in the highest net benefit decreases from 1.00 at $0 per DALY averted to 0.63 at $890 per DALY averted (Fig 1d). The optimal strategy is then routine vaccination with a catch-up campaign with increasing probability from 0.33 at $900 per DALY averted to 0.36 at $1000 per DALY averted. In Scenario 3 (post-outbreak), the probability that no vaccination is the optimal strategy decreases from 0.82 at $0 per DALY averted to 0.53 at $140 per DALY averted. Then, our certainty that routine vaccination with a catchup campaign is preferred increases from 0.33 at a WTP of $150 per DALY averted to 0.63 at $1000 per DALY averted (Fig 1f).

***S1.4.1.*** *Identifying the main drivers of uncertainty.*

The expected value of partially perfect information (EVPPI) is the maximum willingness to pay for additional research regarding a specified parameter, estimated with the one-level method devised by Strong and Oakley [30]. Parameters with the highest EVPPI numbers contribute the most to uncertainty for a particular scenario and across a range of WTP values [30]. For each cost-effectiveness analysis and each uncertain parameter, we estimated the EVPPI (Fig S10-S12).

**S2 Additional Results**

***S2.1.* Predicted and observed typhoid fever infections.**

**
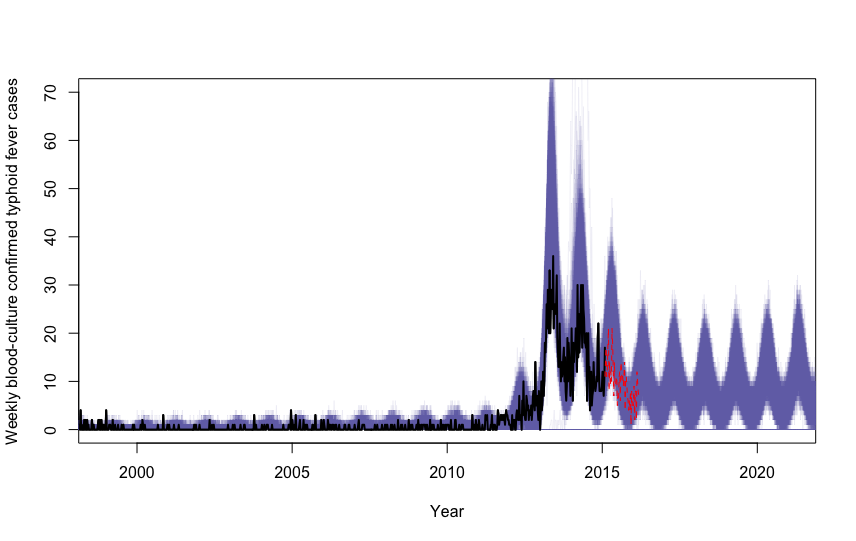
**

**Fig S6. Predicted and observed weekly blood-culture confirmed typhoid fever cases in the absence of vaccination.** The 1,000 stochastic realizations of weekly blood-culture confirmed typhoid fever case incidence per 100,000 people in the absence of vaccination from the dynamic transmission model are show in purple. The observed (reported) typhoid fever cases used to fit the dynamic model is represented by the bold black line, while the observed incidence collected after model fitting is represented by the dashed red line.

**
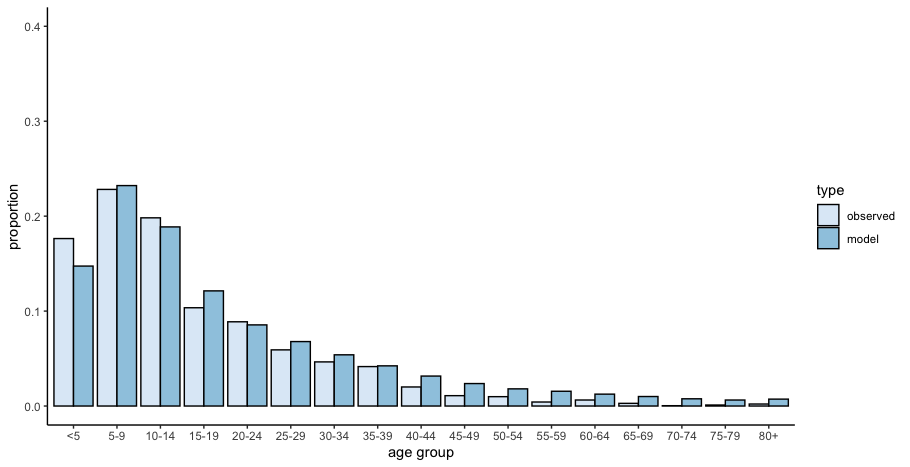
**

**Fig S7. Observed versus fitted age distribution of reported typhoid cases.** The proportion of observed cases in each age group are denoted by light blue bars, while the model-predicted age distribution is shown in darker blue.


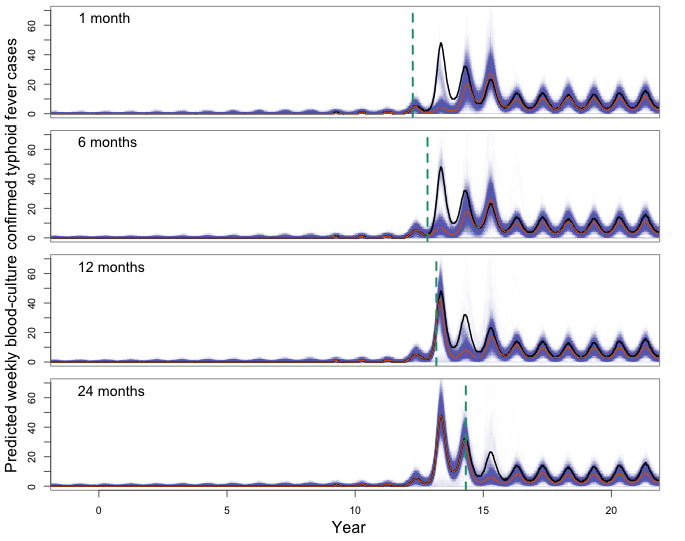


**Fig S8. Predicted weekly blood-culture confirmed typhoid infections in Scenario 1 for reactive vaccination strategies.** The 1,000 simulated predictions for weekly blood-culture confirmed typhoid infections are shown in purple, with the median of all stochastic realizations shown in orange for each reactive vaccination strategy. Four situations are shown, representing the four different delays in timing to implement vaccination once the outbreak is identified (1, 6, 12, and 24 months). The median typhoid infections in the absence of vaccination from 1,000 realizations is shown in black, and the median date of vaccination deployment for each situation is denoted by the vertical dashed green line.


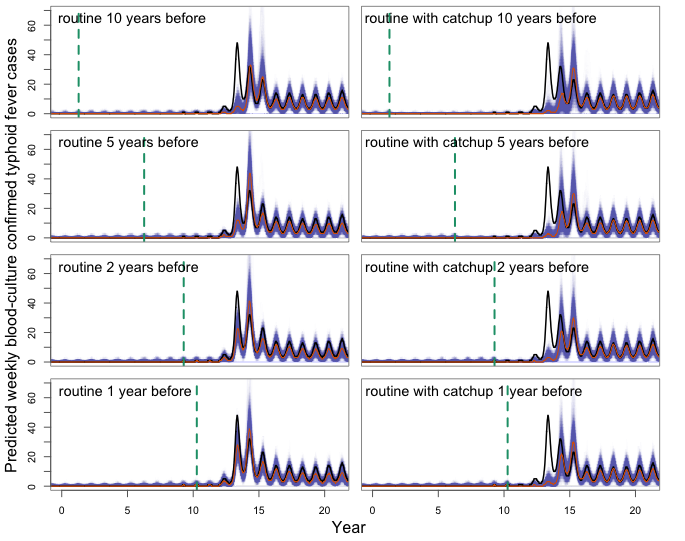


**Fig S9. Predicted weekly blood-culture confirmed typhoid fever cases in Scenario 1 for preventive vaccination strategies.** The 1,000 stochastic realizations of weekly typhoid cases are shown in purple, with the median of all simulations shown in orange for each preventive vaccination strategy. Eight situations are shown, representing each preventive routine vaccination timing strategy (10, 5, 2, and 1 year(s) before the outbreak for routine vaccination at 9 months of age with and without a catchup campaign up to 15 years of age). The median number of typhoid infections in the absence of vaccination from 1,000 realizations is shown in black, and the date of vaccination deployment for each situation is denoted by the vertical dashed green line.

**Table S3. Predicted disease and economic burden in the absence of vaccination.** The median (95% credible interval) estimates for predicted cases, deaths, DALYs, and treatment costs per 100,000 people over 10 years are shown for each scenario in the absence of vaccination.

| **Scenario** | Cases per 100,000 people | Deaths per 100,000 people | DALYs per 100,000 people | Treatment Ccsts per 100,000 people |
| --- | --- | --- | --- | --- |
| **Scenario 1: Randomized outbreak timing** | 1,989 (210 – 3,508) | 18 (1 - 145) | 398 (21 – 3,109) | 126,754 (9,267 – 290,227) |
| **Scenario 2: pre-outbreak incidence** | 146 (73 - 258) | 1 (0 - 8) | 26 (3 - 179) | 5,893 (2,781 – 16,854) |
| **Scenario 3: post-outbreak incidence** | 1,596 (1,169 – 2,631) | 16 (2 - 112) | 402 (56 – 2,751) | 91,891 (49,427 – 191,006) |

**Table S4. Predicted vaccine impact with randomized outbreak timing, pre-outbreak incidence and post-outbreak incidence per 100,000 individuals.** The median (95% credible interval) estimates for averted cases, deaths, DALYs, treatment costs, costs of vaccinations and net costs per 100,000 people are shown for each vaccination strategy compared to no vaccination for randomized outbreak timing (“randomized”), pre-outbreak incidence (“pre”), and post-outbreak incidence (“post”). Vaccination strategies include routine vaccination at nine months of age in year 0 with or without a catchup campaign up to 15 years of age. Reactive routine vaccination strategies (with a catchup campaign) include delays of 1, 6, 12, and 24 months to deployment (“1m”, “6m”, “12m”, “24m”, respectively). All values are presented as incidence per 100,000 people. R=routine vaccination; RC=routine vaccination plus a catchup campaign.

|  | **Strategy** | **Cases averted per 100,000 people** | **Deaths averted per 100,000 people** | **DALYs averted per 100,000 people** | **Averted treatment costs**  **per 100,000 people** | **Cost of vaccination per 100,000 people** | **Net costs per 100,000 people** |
| --- | --- | --- | --- | --- | --- | --- | --- |
| **randomized** | **preventive R** | 485 (-559 - 1,966) | 4 (-9 - 59) | 105 (-148 - 1,308) | 30,208 (-41,420 - 151,651) | 58,060 (26,676 - 134,940) | 27,789 (-96,642 - 128,307) |
|  | **preventive RC** | 890 (2 - 2,594) | 8 (0 - 82) | 199 (6 - 1,820) | 56,037 (-549 - 195,206) | 63,875 (31,372 - 141,005) | 10,059 (-135,184 - 109,202) |
|  | **reactive 1m RC** | 722 (-167 - 2,244) | 7 (-2 - 71) | 157 (-31 - 1,568) | 45,975 (-9,383 - 174,493) | 33,949 (0 - 94,940) | -6,894 (-134,647 - 71,792) |
|  | **reactive 6m RC** | 671 (-296 - 2,139) | 5 (-3 - 71) | 132 (-63 - 1,538) | 41,338 (-21,213 - 170,236) | 31,822 (0 - 90,772) | -5,078 (-132,803 - 77,717) |
|  | **reactive 12m RC** | 471 (-512 - 2,049) | 4 (-7 - 57) | 92 (-149 - 1,270) | 29,579 (-34,155 - 154,498) | 29,153 (0 - 87,383) | -1,702 (-116,023 - 84,940) |
|  | **reactive 24m RC** | 209 (-909 - 1,903) | 2 (-19 - 50) | 38 (-366 - 1,084) | 12,146 (-66,163 - 140,567) | 24,830 (0 - 81,580) | 2,413 (-102,675 - 104,506) |
| **pre** | **R** | 65 (-32 - 178) | 0 (-0 - 5) | 10 (-6 - 102) | 2,688 (-1,286 - 10,376) | 27,134 (13821 - 61,049) | 24,168 (9,347 - 58,205) |
|  | **RC** | 105 (22 - 218) | 1 (0 - 6) | 18 (2 - 137) | 4,297 (822 - 13,455) | 29,377 (15,892 - 62,623) | 24,645 (9,129 - 58,254) |
| **post** | **R** | 661 (-16 - 1,723) | 6 (-0 - 52) | 151 (-9 - 1,255) | 37,448 (-1,081 - 112,982) | 85,536 (39,125 - 199,163) | 46,942 (-39,051 - 164,901) |
|  | **RC** | 840 (202 - 1,899) | 8 (1 - 64) | 199 (19 - 1,577) | 47,452 (10,052 - 127,617) | 93,729 (46,654 - 208,203) | 44,848 (-45,217 - 164,027) |

**Table S5. Predicted vaccine impact with fixed outbreak timing incidence per 100,000 individuals.** The median (95% credible interval) estimates for averted cases, deaths, DALYs, treatment costs, costs of vaccinations and net costs per 100,000 people are shown for each vaccination strategy compared to no vaccination with fixed outbreak timing. vaccination strategies include routine vaccination at nine months of age in year 0 with or without a catchup campaign up to 15 years of age. Preventive strategy results are shown for 10, 5, 2, and 1 year(s) (“10y”, “5y”, “2y”, “1y”) before the outbreak starts. Reactive routine vaccination strategies (with a catchup campaign) include delays of 1, 6, 12, and 24 months to deployment (“1m”, “6m”, “12m”, “24m”, respectively). All values are presented as incidence per 100,000 people. R=routine vaccination; RC=routine vaccination plus a catchup campaign.

| **Strategy** | **Cases averted per 100,000 people** | **Deaths averted per 100,000 people** | **DALYs averted per 100,000 people** | **Averted treatment costs per 100,000 people** | **Costs of vaccination per 100,000 people** | **Net costs per 100,000 people** |
| --- | --- | --- | --- | --- | --- | --- |
| **10y R** | 375 (-282 - 1,215) | 3 (-4 - 37) | 76 (-41 - 691) | 24,131 (-18,282 - 89,166) | 43,217 (20,043 - 98,814) | 19,669 (-46,422 - 88,701) |
| **10y RC** | 509 (-178 - 1,343) | 5 (-2 - 45) | 100 (-15 - 828) | 31,363 (-12,567 - 102,153) | 44,520 (21,386 - 97,911) | 13,325 (-60,767 - 81,578) |
| **5y R** | 314 (-348 - 1,209) | 3 (-5 - 33) | 60 (-67 - 609) | 19,570 (-23,098 - 86,480) | 36,568 (17,246 - 83,365) | 17,846 (-49,271 - 79,750) |
| **5y RC** | 466 (-185 - 1,336) | 4 (-2 - 42) | 93 (-15 - 808) | 29,081 (-11,629 - 97,161) | 38,772 (19,138 - 85,705) | 10,484 (-59,096 - 71,241) |
| **2y R** | 262 (-483 - 1,149) | 2 (-7 - 30) | 45 (-109 - 554) | 16,381 (-32,486 - 80,460) | 31,340 (14,792 - 70,716) | 16,435 (-48,932 - 75,998) |
| **2y RC** | 431 (-223 - 1,272) | 4 (-2 - 40) | 87 (-19 - 743) | 28,188 (-12,290 - 92,518) | 34,034 (17,274 - 74,542) | 7,421 (-58,806 - 64,972) |
| **1y R** | 239 (-522 - 1,077) | 2 (-10 - 28) | 40 (-144 - 497) | 14,437 (-34,518 - 75,971) | 29,333 (13,824 - 66,362) | 15,751 (-48,606 - 77,849) |
| **1y RC** | 432 (-288 - 1,293) | 4 (-3 - 40) | 85 (-32 - 757) | 27,500 (-17,169 - 95,888) | 32,494 (16,569 - 69,920) | 6,216 (-63,164 - 62,151) |
| **1m RC** | 404 (-181 - 1,246) | 4 (-2 - 38) | 80 (-17 - 715) | 26,515 (-11,647 - 94,671) | 28,307 (14,898 - 61,364) | 2,915 (-64,109 - 53,470) |
| **6m RC** | 383 (-277 - 1,277) | 4 (-3 - 39) | 76 (-37 - 718) | 25,571 (-17,153 - 93,658) | 27,441 (14,297 - 59,357) | 2,985 (-66,026 - 53,385) |
| **12m RC** | 370 (-265 - 1,210) | 4 (-3 - 39) | 71 (-35 - 698) | 23,959 (-15,282 - 89,853) | 26,106 (13,841 - 57,262) | 3,253 (-61,706 - 53,180) |
| **24m RC** | 302 (-465 - 1,112) | 3 (-7 - 32) | 52 (-106 - 561) | 18,969 (-30,239 - 79,331) | 24,327 (13,141 - 51,560) | 6,383 (-54,402 - 61,926) |

**Table S6. Predicted vaccine impact with WHO-CHOICE cost of illness data: randomized outbreak timing, pre-outbreak incidence and post-outbreak incidence.** The median (95% credible interval) estimates for averted treatment costs and net costs per 100,000 people are shown (averted cases, deaths, DALYs, and costs of vaccinations are the same as the main scenarios) for each vaccination strategy compared to no vaccination for randomized outbreak timing (“randomized”), pre-outbreak incidence (“pre”), and post-outbreak incidence (“post”) using previous WHO-CHOICE cost of illness data. Vaccination strategies include routine vaccination at nine months of age in year 0 with or without a catchup campaign up to 15 years of age. Reactive routine vaccination strategies (with a catchup campaign) include delays of 1, 6, 12, and 24 months to deployment (“1m”, “6m”, “12m”, “24m”, respectively). All values are presented as costs per 100,000 people. R=routine vaccination; RC=routine vaccination plus a catchup campaign.

|  | **Strategy** | **Averted treatment costs per 100,000 people** | **Net costs per 100,000 people** |
| --- | --- | --- | --- |
| **randomized** | **preventive R** | 2,255 (-3841 - 17377) | 54,251 (20416 - 129404) |
|  | **preventive RC** | 4,243 (-27 - 23697) | 57,436 (21751 - 133704) |
|  | **reactive 1m RC** | 3,385 (760 - 20862) | 28,051 (-223 - 88410) |
|  | **reactive 6m RC** | 3,034 (-1627 - 19832) | 26,127 (-336 - 85197) |
|  | **reactive 12m RC** | 2,146 (-2602 - 17552) | 24,530 (-750 - 81069) |
|  | **reactive 24m RC** | 912 (-6324 - 14830) | 22,144 (-1935 - 76954) |
| **pre** | **R** | 123 (-63 - 675) | 26,854 (13536 - 59612) |
|  | **RC** | 206 (33 - 935) | 28,940 (15334 - 61745) |
| **post** | **R** | 2,942 (112 - 12989) | 80,862 (32817 - 193073) |
|  | **RC** | 3,635 (285 - 15703) | 88,714 (39969 - 199384) |

**
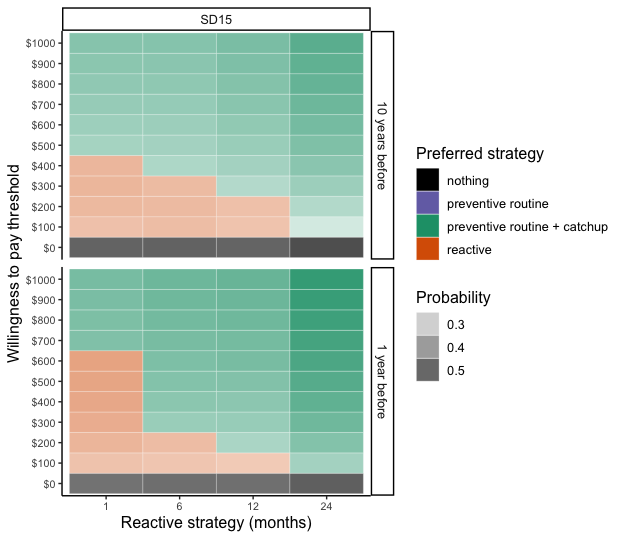
**

**Fig S10. Heatmap of optimal intervention strategy and its estimated uncertainty across a range of willingness to pay values for each strategy comparison and a range of deployment delays, and years before the outbreak.** Each column in a single panel shows the preferred strategy (i.e. the strategy that yields the highest average net benefit) for one cost-effectiveness analysis comparing no vaccination (grey), preventive routine vaccination (purple), preventive routine vaccination with a catch-up campaign (green), and reactive vaccination with a catch-up campaign (orange) for delays of 1, 6, 12, or 24 months after the outbreak has been identified (x-axis). The y-axis represents willingness-to-pay (WTP) values ranging from $0-$1000 (USD 2020). The shading represents the probability that the preferred strategy yields the highest net benefit (lighter: lower probability; darker: higher probability). Results are plotted for whether preventive vaccination is introduced 10 years (top panel) or 1 year (bottom panel) before the outbreak for a 20-year time horizon. Note that preventive routine vaccination without a catchup campaign is never a preferred strategy, and as a result does not appear in the plots.

**
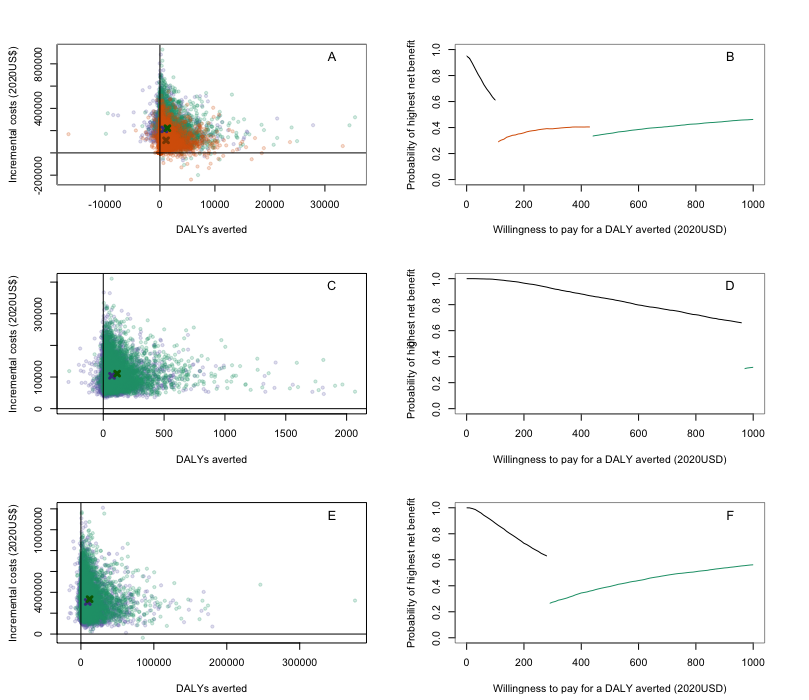
**

**Fig S11. Cost-effectiveness planes and acceptability frontiers for sensitivity analysis assuming WHO-CHOICE treatment costs.** The cost-effectiveness planes (left) and cost-effectiveness acceptability frontiers (CEAFs; right) are plotted for (A-B) Scenario 1 (randomized outbreak timing), (C-D) Scenario 2 (no outbreak, assuming pre-outbreak incidence), and (E-F) Scenario 3 (outbreak has already occurred). In the cost-effectiveness planes, each dot represents the incremental costs (in 2020 USD) and DALYs averted for one simulation when compared with the base case strategy of no vaccination. The bold Xs denote the expected additional cost and DALYs averted for each vaccination strategy with respect to no vaccination. Strategies are indicated by the color of the dot or X (purple: preventive routine vaccination; green: preventive routine vaccination plus a catch-up campaign up to age 15; or orange: reactive routine vaccination plus a catch-up campaign to age 15—for Scenario 1 only). In the CEAFs, the preferred strategy (i.e. the strategy that yielded the highest *average* net benefit) for each willingness-to-pay threshold ($0-1,000 per DALY averted; x-axis, 2020 USD) is indicated by the color of the line (black: no vaccination; and same strategy colors as other panels), while the proportion of samples in which that strategy yielded the highest net benefit is indicated by the value on the y-axis; this can be interpreted as our certainty in the optimal strategy.


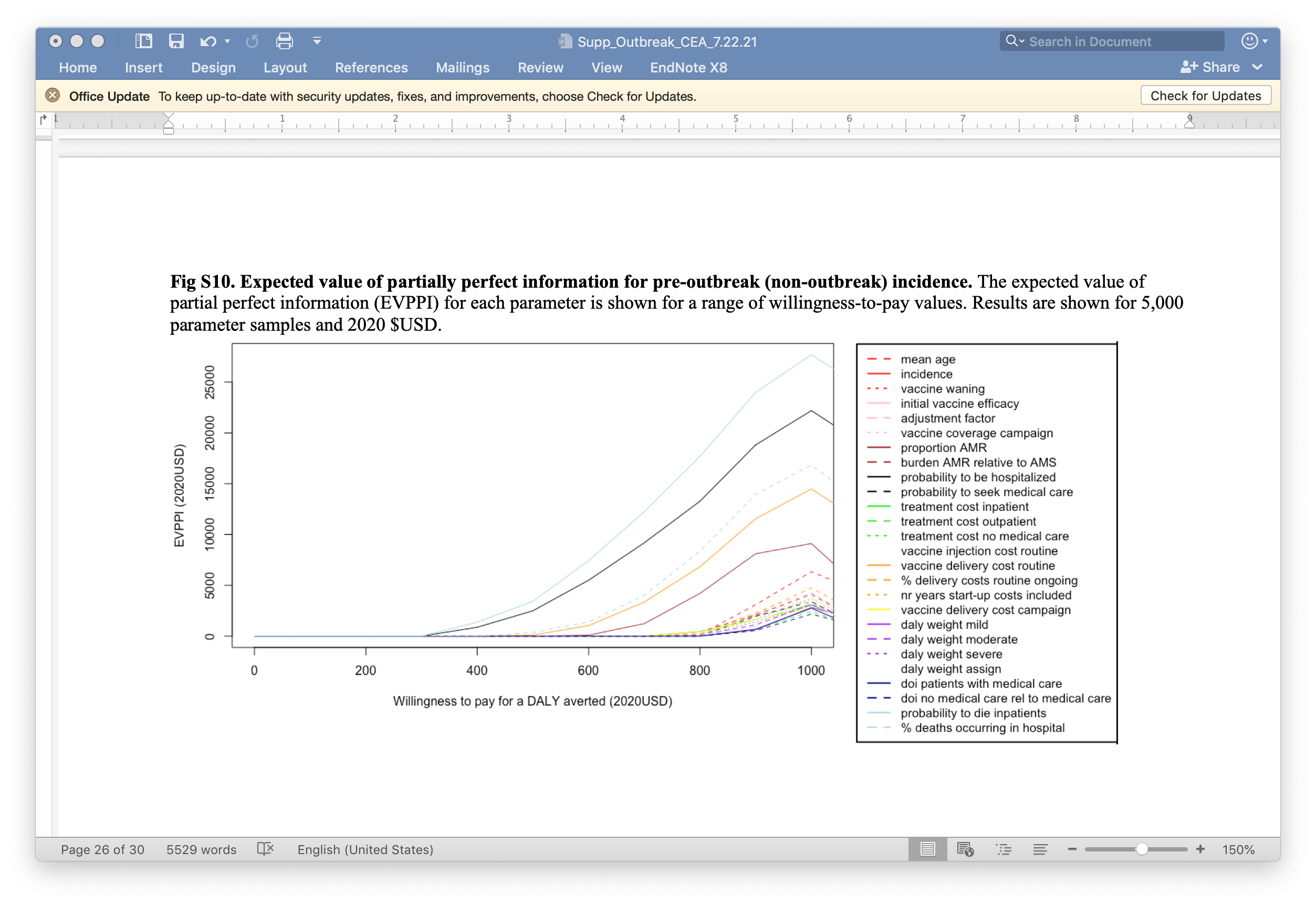

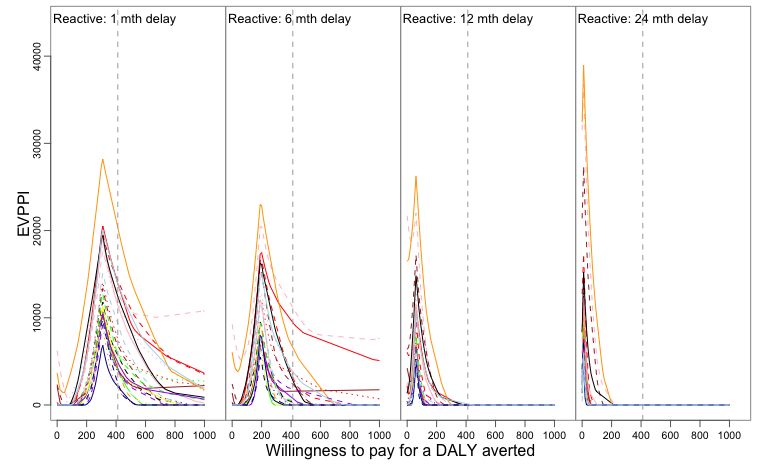


**Fig S12. Expected value of partially perfect information for differing delays in vaccination deployment for reactive strategies with randomized outbreak timing (Scenario 1).** The expected value of partial perfect information (EVPPI) for each parameter is shown for a range of willingness-to-pay values. Results are shown for 5,000 parameter samples (in 2020 USD). Each panel shown represents the EVPPI for one cost-effectiveness analysis comparing 4 strategies: no vaccination (base case), preventive routine vaccination at 9 months, preventive routine vaccination with a catch-up campaign up to 15 years, and reactive routine vaccination with a catch-up campaign. The four panels (left to right) correspond to different delays in the reactive strategy (1-, 6-, 12-, or 24-month delays). The grey vertical dashed line corresponds to the 2020 gross domestic product per capita for Malawi.


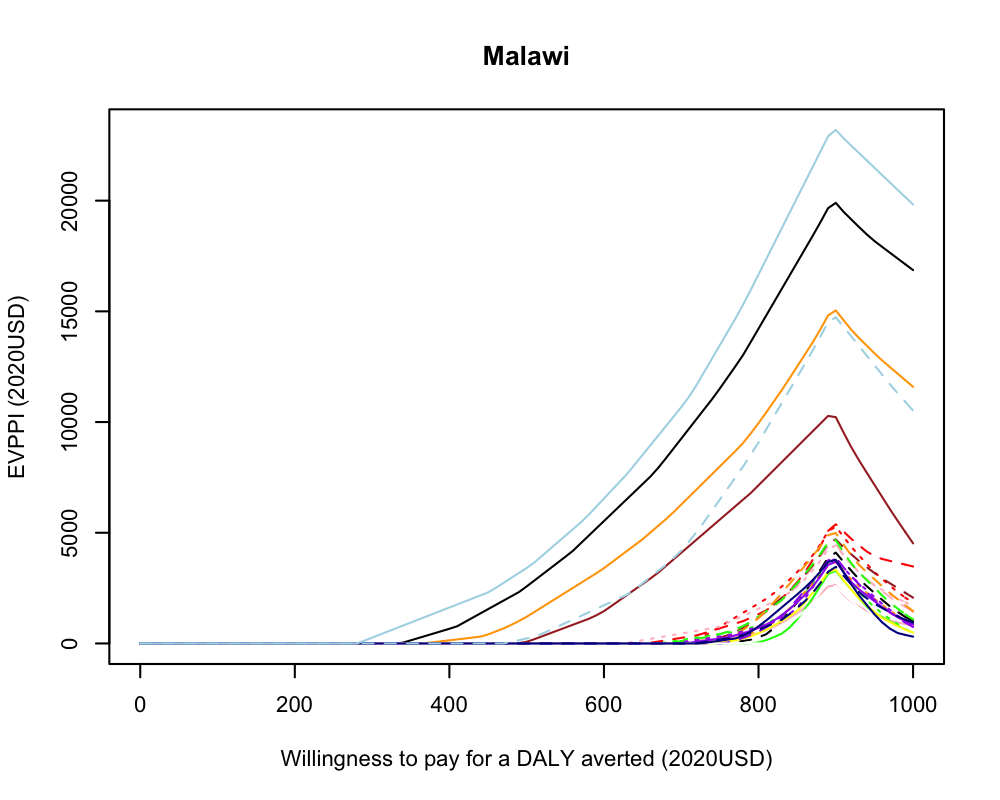

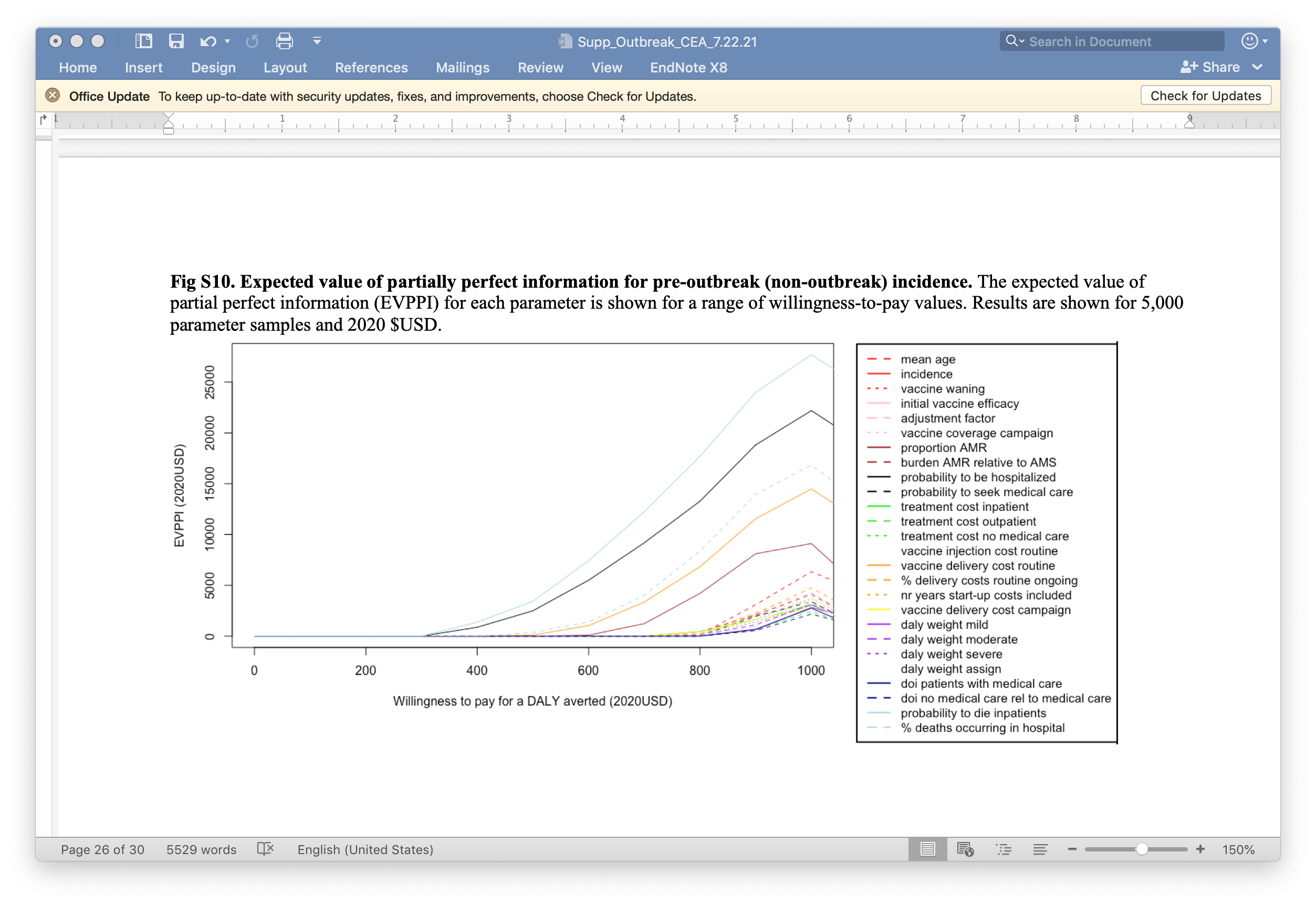


**Fig S13. Expected value of partially perfect information for Scenario 2 (pre-outbreak incidence).** The expected value of partial perfect information (EVPPI) for each parameter is shown for a range of willingness-to-pay values. Results are shown for 5,000 parameter samples in 2020 USD.

**
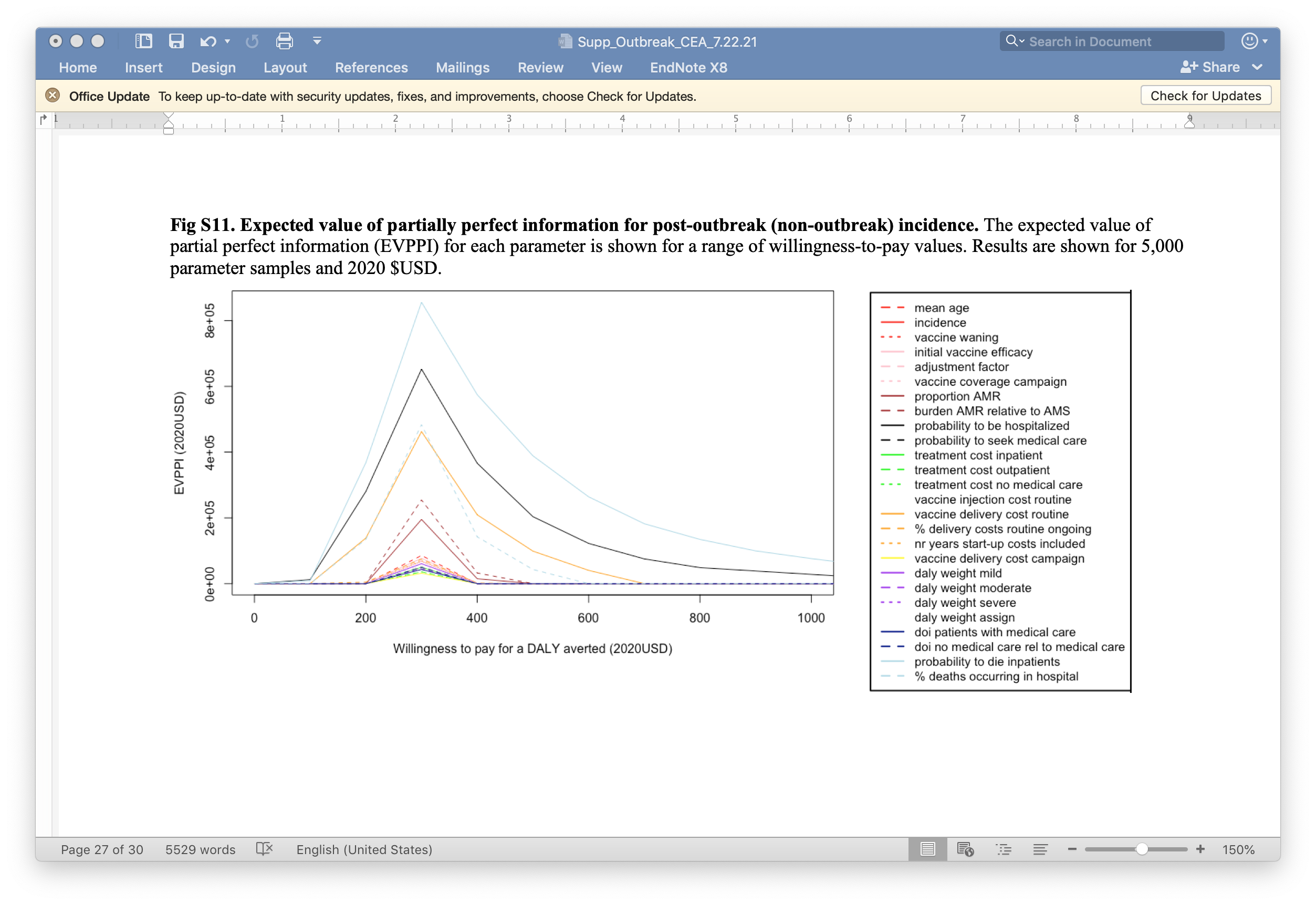
**


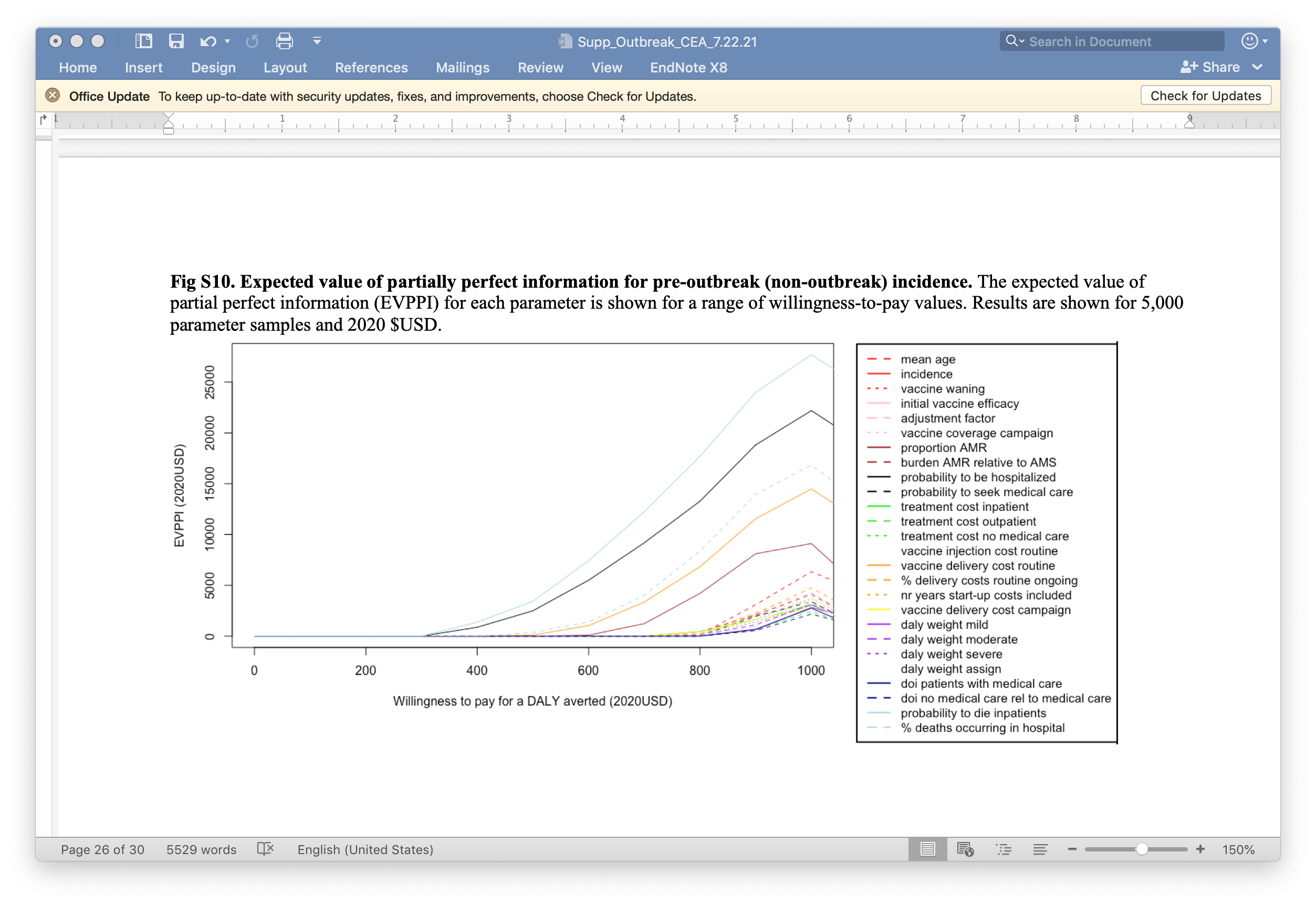

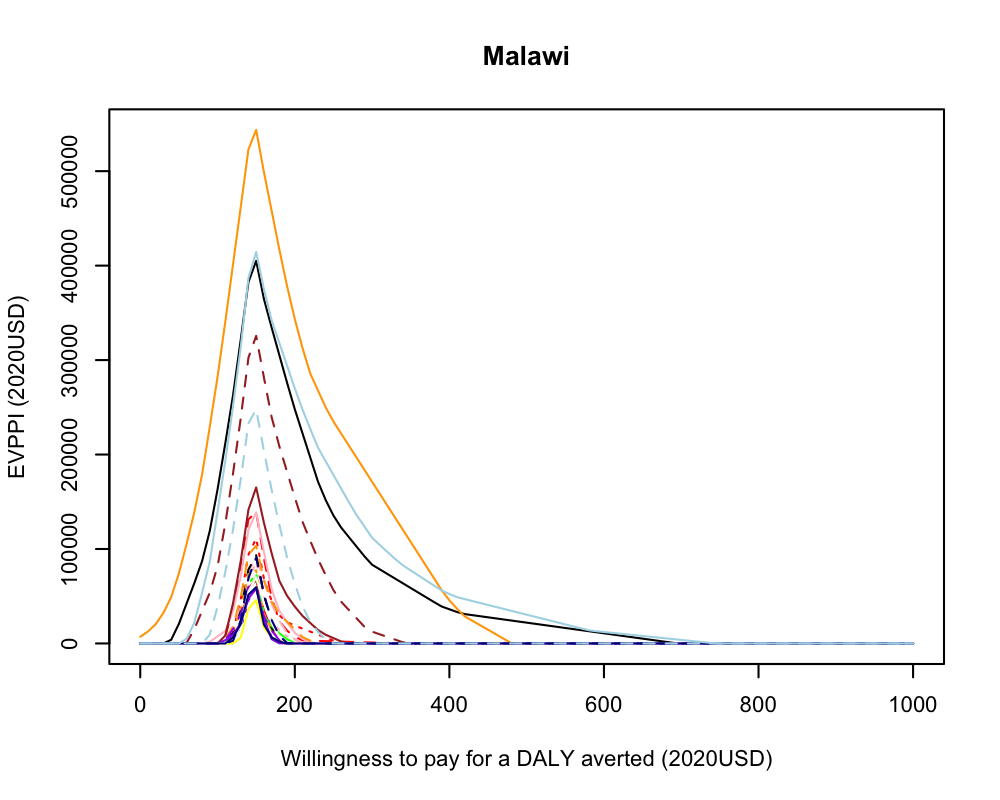


**Fig S14. Expected value of partially perfect information for Scenario 3 (post-outbreak incidence).** The expected value of partial perfect information (EVPPI) for each parameter is shown for a range of willingness-to-pay values. Results are shown for 5,000 parameter samples in 2020 USD.

***S3.* Consolidated Health Economics Evaluation Reporting Standards (**CHEERS) **checklist**


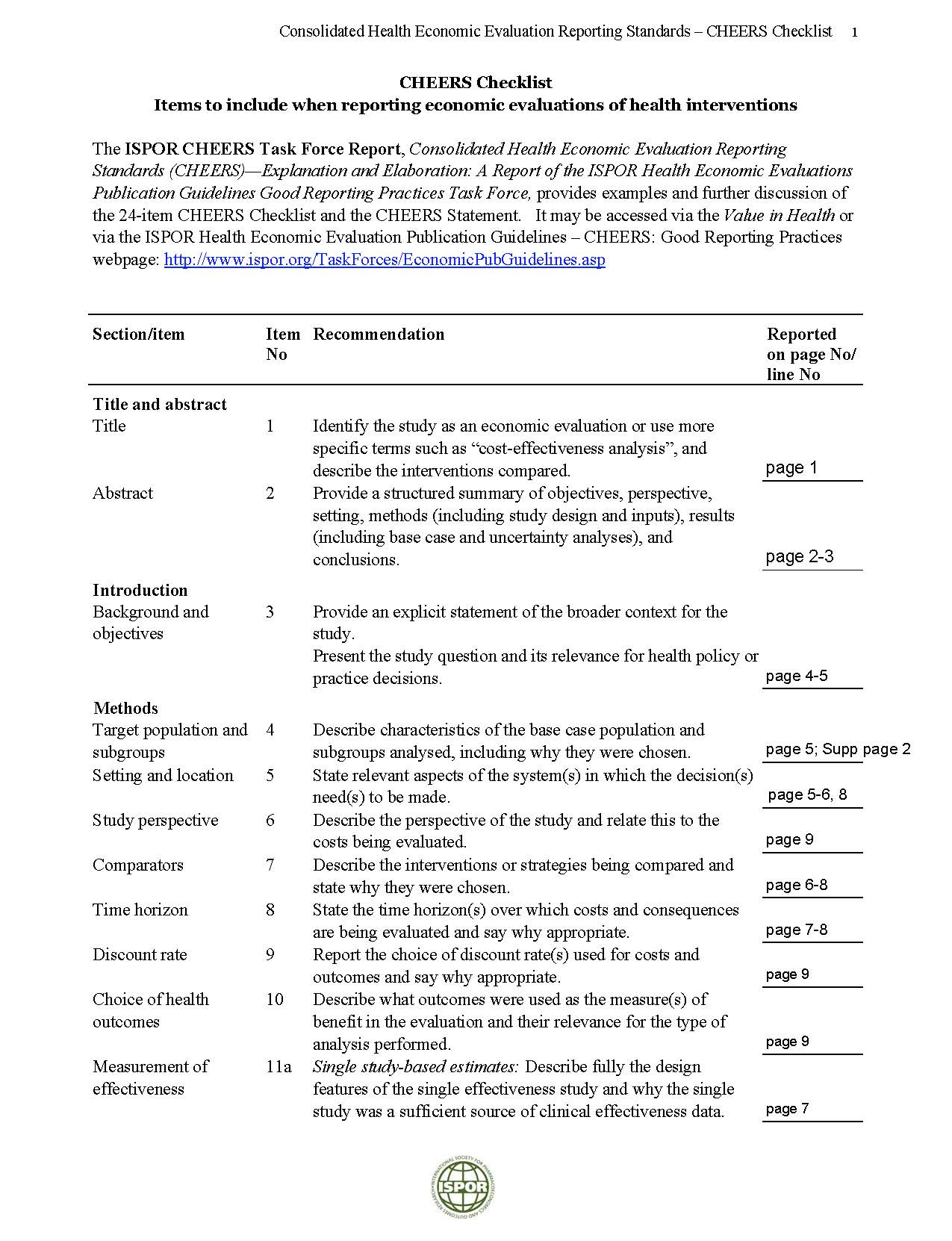


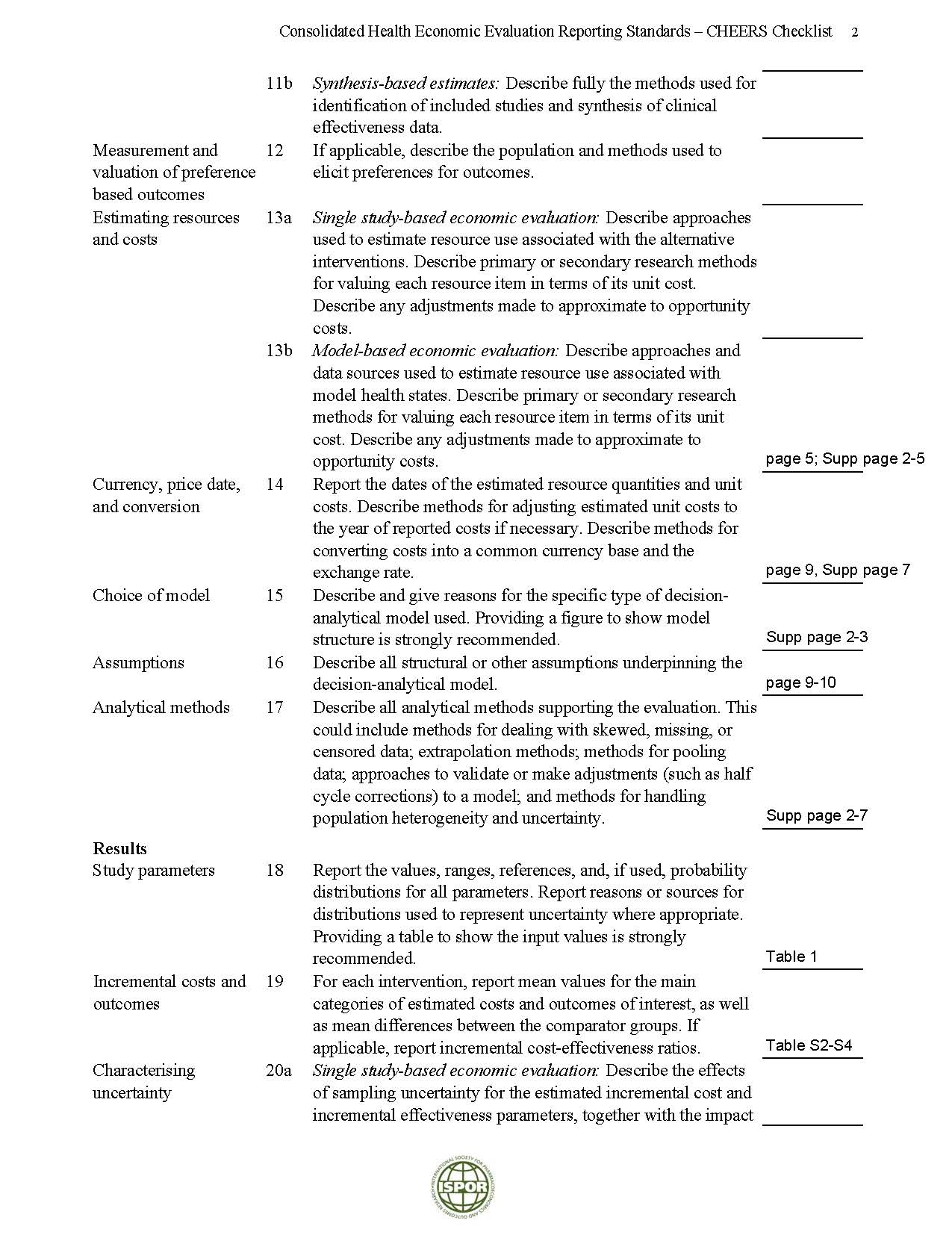

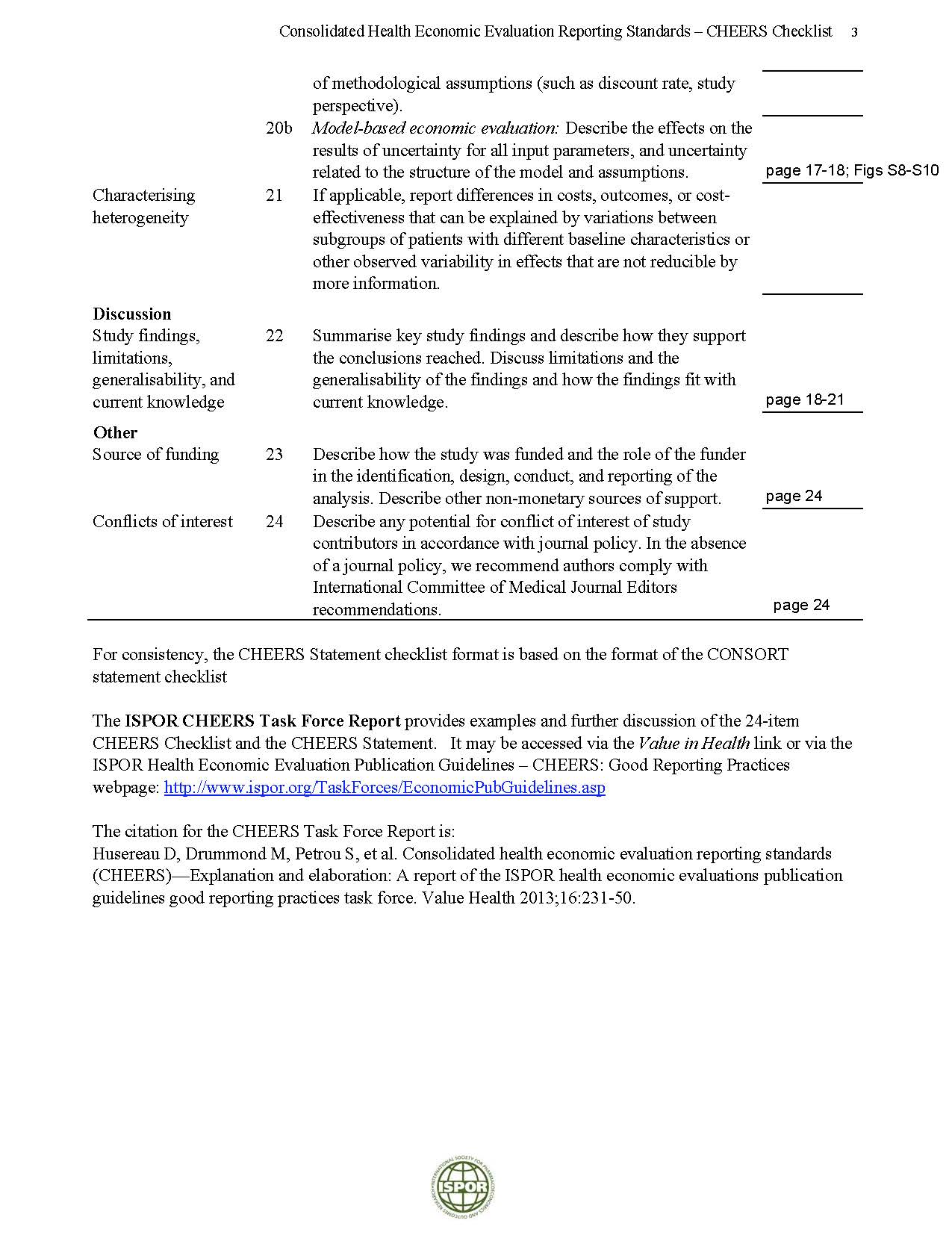


References

1. Ames WR, Robins M. Age and Sex as Factors in the Development of the Typhoid Carrier State, and a Method for Estimating Carrier Prevalence *. Am J Public Health Nations Health. 1943;33(3):221-30. PubMed PMID: 18015749; PubMed Central PMCID: PMC1527221.

2. Phillips MT, Meiring JE, Voysey M, Warren JL, Baker S, Basnyat B, et al. A Bayesian approach for estimating typhoid fever incidence from large-scale facility-based passive surveillance data. Statistics in Medicine. 2021;40(26):5853-70. doi: 10.1002/sim.9159.

3. Feasey N, Gaskell K, Wong V, Msefula C, Selemani G, Kumwenda S, et al. Rapid Emergence of Multidrug Resistant, H58-Lineage Salmonella Typhi in Blantyre, Malawi. PLOS Neglected Tropical Diseases. 2015;9(4):e0003748. doi: 10.1371/journal.pntd.0003748.

4. Pitzer VE, Feasey NA, Msefula C, Mallewa J, Kennedy N, Dube Q, et al. Mathematical Modeling to Assess the Drivers of the Recent Emergence of Typhoid Fever in Blantyre, Malawi. Clinical infectious diseases : an official publication of the Infectious Diseases Society of America. 2015;61:S251-8. Epub 2015/10/10. doi: 10.1093/cid/civ710. PubMed PMID: 26449939; PubMed Central PMCID: PMCPMC4596932.

5. Bilcke J, Antillon M, Pieters Z, Kuylen E, Abboud L, Neuzil KM, et al. Cost-effectiveness of routine and campaign use of typhoid Vi-conjugate vaccine in Gavi-eligible countries: a modelling study - The Lancet Infectious Diseases. Lancet Infectious Diseases. 2019;19(7):728-39. doi: doi:10.1016/S1473-3099(18)30804-1.

6. Gibani M, Voysey M, Jin C, Jones C, Thomaides-Brears H, E J, et al. The Impact of Vaccination and Prior Exposure on Stool Shedding of Salmonella Typhi and Salmonella Paratyphi in 6 Controlled Human Infection Studies. Clinical infectious diseases. 2019;68(8). doi: 10.1093/cid/ciy670. PubMed PMID: 30252031.

7. Sur D, Ochiai RL, Bhattacharya SK, Ganguly NK, Ali M, Manna B, et al. A Cluster-Randomized Effectiveness Trial of Vi Typhoid Vaccine in India. NEJM. 2009;361(4). doi: 10.1056/NEJMoa0807521.

8. Voysey M, Pollard A. Seroefficacy of Vi Polysaccharide-Tetanus Toxoid Typhoid Conjugate Vaccine (Typbar TCV). Clinical infectious diseases : an official publication of the Infectious Diseases Society of America. 2018;67(1). doi: 10.1093/cid/cix1145. PubMed PMID: 29351594.

9. Jin C, Gibani M, Moore M, Juel H, Jones E, Meiring J, et al. Efficacy and immunogenicity of a Vi-tetanus toxoid conjugate vaccine in the prevention of typhoid fever using a controlled human infection model of Salmonella Typhi: a randomised controlled, phase 2b trial. Lancet (London, England). 2017;390(10111). doi: 10.1016/S0140-6736(17)32149-9. PubMed PMID: 28965718.

10. Mohan V, Varanasi V, Singh A, Pasetti M, Levine M, Venkatesan R, et al. Safety and immunogenicity of a Vi polysaccharide-tetanus toxoid conjugate vaccine (Typbar-TCV) in healthy infants, children, and adults in typhoid endemic areas: a multicenter, 2-cohort, open-label, double-blind, randomized controlled phase 3 study. Clinical infectious diseases : an official publication of the Infectious Diseases Society of America. 2015;61(3). doi: 10.1093/cid/civ295. PubMed PMID: 25870324.

11. Patel P, Patel P, Liang Y, Meiring J, Misiri T, Mwakiseghile F, et al. Safety and Efficacy of a Typhoid Conjugate Vaccine in Malawian Children. The New England journal of medicine. 2021;385(12). doi: 10.1056/NEJMoa2035916. PubMed PMID: 34525285.

12. Shakya M, Voysey M, Theiss-Nyland K, Colin-Jones R, Pant D, al e. Efficacy of typhoid conjugate vaccine in Nepal: final results of a phase 3, randomised, controlled trial - The Lancet Global Health. Lancet Global Health. 2021;9(11):E1561-8. doi: 0.1016/S2214-109X(21)00346-6.

13. Qadri F, Khanam F, Liu X, Liu, Theiss-Nyland K, Biswas PK, Bhuiyan AI. Protection by vaccination of children against typhoid fever with a Vi-tetanus toxoid conjugate vaccine in urban Bangladesh: a cluster-randomised trial - The Lancet. Lancet (London, England). 2021;398(10301):675-84. doi: 10.1016/S0140-6736(21)01124-7.

14. Meiring J, Laurens M, Patel P, al e. Typhoid vaccine acceleration consortium Malawi: A Phase III, randomized, double-blind, controlled trial of the clinical efficacy of typhoid conjugate vaccine among children in Blantyre, Malawi. Clinical Infectious Diseases. 2019;68:S50-8.

15. Meiring J, Sambakunsi R, Moyo E, al e. Community engagement before initiation of typhoid conjugate vaccine trial in schools in two urban townships in Blantyre, Malawi: Experience and lessons. Clinical Infectious Diseases. 2019;68:S146-53.

16. Sur D, Barkume C, Mukhopadhyay B, Date K, Ganguly NK, Garrett D. A Retrospective Review of Hospital-Based Data on Enteric Fever in India, 2014-2015. J Infect Dis. 2018. Epub 2018/10/12. doi: 10.1093/infdis/jiy502. PubMed PMID: 30307566.

17. Antillon M, Bilcke J, Paltiel AD, Pitzer VE. Cost-effectiveness analysis of typhoid conjugate vaccines in five endemic low- and middle-income settings. Vaccine. 2017;35(27):3506-14. Epub 2017/05/22. doi: 10.1016/j.vaccine.2017.05.001. PubMed PMID: 28527687; PubMed Central PMCID: PMCPMC5462484.

18. Stenberg K, Lauer JA, Gkountouras G, Fitzpatrick C, Stanciole A. Econometric estimation of WHO-CHOICE country-specific costs for inpatient and outpatient health service delivery. Cost Effectiveness and Resource Allocation. 2018;16(1):1-15. doi: 10.1186/s12962-018-0095-x.

19. McMahon T. Historical Consumer Price Index (CPI): Capital Professional Services; 2021 [cited 2021 May 11]. Available from: <https://inflationdata.com/Inflation/Consumer_Price_Index/HistoricalCPI.aspx?reloaded=true#Table?reloaded=true?reloaded=true>.

20. Gandhi G, Lydon P. Updating the evidence base on the operational costs of supplementary immunization activities for current and future accelerated disease control, elimination and eradication efforts. BMC Public Health. 2014;14(1):1-16. doi: doi:10.1186/1471-2458-14-67.

21. Limani F, Smith C, Wachepa R, Chafuwa H, Meiring J, Noah P, et al. Estimating the economic burden of typhoid in children and adults in Blantyre, Malawi: a costing cohort study. Research Square. 2021. doi: 10.21203/rs.3.rs-1214074/v1.

22. Global Burden of Disease Study. Global Burden of Disease Study 2010 (GBD 2010) Results by Cause 1990-2010 Seattle, United States: Institute for Health Metrics and Evaluation (IHME); 2012. Available from: <http://ghdx.healthdata.org/record/global-burden-disease-study-2010-gbd-2010-results-cause-1990-2010>.

23. Salomon JA, Vos T, Hogan DR, Gagnon M, Naghavi M, Mokdad A, et al. Common values in assessing health outcomes from disease and injury: disability weights measurement study for the Global Burden of Disease Study 2010. Lancet (London, England). 2012;380(9859):2129-43. Epub 2012/12/19. doi: 10.1016/s0140-6736(12)61680-8. PubMed PMID: 23245605.

24. Darton TC, Meiring JE, Tonks S, Khan MA, Khanam F, Shakya M, et al. The STRATAA study protocol: a programme to assess the burden of enteric fever in Bangladesh, Malawi and Nepal using prospective population census, passive surveillance, serological studies and healthcare utilisation surveys. 2017;7(6). doi: 10.1136/bmjopen-2017-016283.

25. Abboud L. Global hospitalization rate of typhoid fever: a meta-regression. Antwerp: University of Antwerp; 2017.

26. Pieters Z, Saad NJ, Antillón M, Pitzer VE, Bilcke J. Case Fatality Rate of Enteric Fever in Endemic Countries: A Systematic Review and Meta-analysis. Clinical Infectious Diseases. 2018;67(4):628-38. doi: 10.1093/cid/ciy190.

27. Marchello CS, Birkhold M, Crump JA. Complications and mortality of typhoid fever: A global systematic review and meta-analysis. Journal of Infection. 2020;81(6):902-10. doi: 10.1016/j.jinf.2020.10.030.

28. World Bank. Life expectancy at birth, total (years) 2021. 2019 Revision:[Available from: <https://data.worldbank.org/indicator/SP.DYN.LE00.IN>.

29. World Bank. GDP per capita (current US$) - Malawi: The World Bank Group; 2021 [cited 2021 May 11]. Available from: <https://data.worldbank.org/indicator/NY.GDP.PCAP.CD?locations=MW>.

30. Strong MO, Jeremy E. An Efficient Method for Computing Single-Parameter Partial Expected Value of Perfect Information. Medical Decision Making. 2012;33(6):755-66. doi: 10.1177_0272989X12465123.
